# Comparison of the upper and lower airway microbiota in children with chronic lung diseases

**DOI:** 10.1371/journal.pone.0201156

**Published:** 2018-08-02

**Authors:** Bushra Ahmed, Michael J. Cox, Leah Cuthbertson, Phillip L. James, William O. C. Cookson, Jane C. Davies, Miriam F. Moffatt, Andrew Bush

**Affiliations:** 1 National Heart and Lung Institute, Imperial College London, London, United Kingdom; 2 Department of Respiratory Paediatrics, Royal Brompton Hospital, London, United Kingdom; Telethon Institute for Child Health Research, AUSTRALIA

## Abstract

**Rationale:**

The lower airway microbiota is important in normal immunological development and chronic lung diseases (CLDs). Young children cannot expectorate and because of the uncertainty whether upper airway samples reflect the lower airway microbiota, there have been few longitudinal paediatric studies to date.

**Objectives:**

To assess whether throat swabs (TS) and cough swabs (CS) are representative of the lower airway microbiota.

**Methods:**

TS, CS, bronchoalveolar lavage and bronchial brushings were prospectively collected from 49 children undergoing fibreoptic bronchoscopy for CLDs. Bacterial DNA was extracted and the 16S rRNA gene V4 region sequenced using the Illumina MiSeq.

**Results:**

5.97 million high quality reads were obtained from 168 samples (47 TS, 37 CS, 42 BALF and 42 bronchial brushings). CS sequenced poorly. At a community level, no difference in alpha diversity (richness, evenness or Shannon Diversity Index) was seen between lower airway samples and TS (*P* > 0.05). Less than 6.31% of beta diversity variation related to sampling method for TS (*P* = 0.001). Variation between pathologies and individual patients was greater (20%, 54% respectively *P* ≤ 0.001) than between TS and lower airway samples. There was strong correlation in the relative abundance of genera between samples (r = 0.78, *P* < 0.001). Similarity between upper and lower airway samples was observed to be less for individuals where one sample type was dominated by a single organism.

**Conclusions:**

At the community structure level, TS correlate with lower airway samples and distinguish between different CLDs. TS may be a useful sample for the study of the differences in longitudinal changes in the respiratory microbiota between different CLDs. Differences are too great however for TS to be used for clinical decision making.

## Introduction

Complex microbial communities inhabit the healthy lower airways, that were once thought to be sterile, and are important in chronic lung diseases such as cystic fibrosis (CF)[1]. Using molecular techniques, chronic suppurative airway diseases are now believed to be more polymicrobial than previously appreciated[2–4] with increasing interest in the role the microbiota has to play in shaping normal and pathological airway immune responses.

Whilst studies in adults have highlighted the importance of the microbiota in disease progression in CF, comparatively less is known about the early development of the airway microbiota in children. This is in part because longitudinal study of the airway microbiota in children is challenging due to the difficulties in obtaining repeated lower airway samples. Most children cannot spontaneously expectorate and obtaining bronchoalveolar lavage fluid (BALF) frequently is neither ethical nor feasible. In the only two longitudinal studies of the infant lower airway microbiota reported to date, BALF was collected infrequently (at most every 6 months)[5,6] thus limiting the utility of BALF to track concurrent changes in the microbiota with symptoms. Nasopharyngeal sampling has also been used to track monthly changes in the airway microbiota in infants[7–9], but this has been shown to be a poor surrogate for the lower airway microbiota[10]. Thus, there is a need to identify a reliable surrogate for lower airway sampling which can be collected frequently and is well tolerated by children.

In clinical practice, cultures of either throat swabs (TS) or cough swabs (CS) are regularly used as surrogates for lower airway samples. Culture dependent results of TS and CS are however unreliable due to variability in sensitivity when compared with lower airway results: e.g. conventional culture sensitivity for *Pseudomonas aeruginosa* ranges from 35.7–71%[11,12]. Whether the poor sensitivity of upper airway samples is due to the limitations of microbial cultures or the sampling method however remains unclear.

Similarities in the microbiota between throat swabs and lower airway samples have been reported in older expectorating children with CF (TS compared with sputum)[10] as well as in adults (N = 6 adults, TS compared with BALF)[13]. In younger children and infants with chronic lung diseases, the use of upper airway samples, particularly CS, has not been validated and was the objective of the present study. We hypothesised that upper airway samples (CS and TS) would be a reliable surrogate for the lower airway microbiota. We aimed to compare TS and CS with lower airway samples obtained at bronchoscopy in children with CLDs undergoing a clinically indicated procedure.

## Methods

For additional details please see S1 Appendix.

### Subjects and sampling

Children undergoing a clinical bronchoscopy at the Royal Brompton Hospital (RBH) between December 2012 and May 2013 were recruited. Ethical approval was granted by the RBH NIHR Biomedical Research Unit Advanced Lung Disease Biobank (NRES reference 10/H0504/9). Parental written consent and age-appropriate assent from the child was obtained.

At least one upper airway (TS or CS) and a paired lower airway sample (BALF or bronchial brushing) were collected from each child (see S1 Appendix). Bacterial culture of BALF was performed as per standard clinical practice.

### 16S rRNA gene library preparation and sequencing

A maximum of 2 x 2ml aliquots of BALF (median 3.2ml, range 0.5–4ml) were centrifuged at 21,000g for 30 minutes and the cell pellet retained for DNA extraction. Frozen swab and brushing heads were transferred directly into a Lysing Matrix E tube containing sodium phosphate buffer. DNA was extracted using the MP Bio FastDNA Spin Kit for Soil (http://www.mpbio.com) according to the manufacturer’s instructions.

Quadruplicate PCRs of the 16S rRNA gene V4 region were performed using a custom indexed forward primer S-D-Bact-0564-a-S-15 (5’ AYT GGG YDT AAA GNG 3’), reverse primer S-D-Bact-0785-b-A-18 (5’ TAC NVG GGT ATC TAA TCC 3’)[14] and a high fidelity *Taq* polymerase master mix (Q5, New England Biolabs). A mock community was included to assess sequencing quality. PCR cycling conditions were: annealing at 95°C for 2 minutes followed by 35 cycles at 95°C for 20 seconds, 50°C for 20 seconds and 72°C for 5 minutes. Amplicons were purified, quantified and equi-molar pooled to form a DNA library for paired-end sequencing using the Illumina MiSeq V2 reagent kit [15] (S1 Fig for laboratory workflow). With the exception of 10 samples (details given in S1 Appendix), all samples from an individual patient were run on the same plate to limit the impact of any batch effect.

### Data analysis

Sample size was opportunistic in the absence of data to inform a power calculation at the inception of the study. Upstream processing was performed using Quantitative Insights into Microbial Ecology (QIIME) Version 1.9.0 (see S1 Appendix). Downstream analyses, to assess community level differences in diversity and Operational Taxonomic Unit (OTU) level differences, were performed using Phyloseq in R version 3.2.0 (S2 Fig). Within samples, alpha-diversity differences in richness (the number of different species), evenness (the spread of species) and the Shannon Diversity Index were calculated using paired t-tests and Wilcoxon sign-ranked tests for parametric and non-parametric data respectively. The Shannon Diversity Index is a composite measure of richness and evenness which quantifies the uncertainty in predicting species identity when randomly sampling from a community. Bland-Altman plots were constructed to analyse the agreement in alpha diversity between samples.

Between sample, beta-diversity differences were tested using the Bray Curtis dissimilarity, the unweighted UniFrac and weighted UniFrac scores using a permutational multivariate ANOVA (PERMANOVA)[16], in which the r^2^ value represents the degree of variance in community composition explained by the variables tested in the model. Block designs were used for paired comparisons between samples from the same patient. The Bray-Curtis index measures dissimilarity between two samples by estimating the number of shared organisms from the total number of organisms present in both samples; the higher the Bray Curtis dissimilarity score, the greater the difference between two samples. The UniFrac score is a qualitative measure of the phylogenetic distance between two communities, with the weighted UniFrac score additionally accounting for the relative abundance of organisms within these samples. Quantitative measures of beta diversity, such as Bray Curtis dissimilarity and weighted UniFrac scores, provide information on community differences due to the relative abundance of species. The UniFrac score provides information on community differences driven by selective pressures on the community[17].

OTU level differences were assessed using Spearman’s rank correlation. The Multtest package in R was used to perform multiple t-tests with Benjamini-Hochberg correction to compare differences in the relative abundance of genera between upper and lower airway samples. A *P* value of less than 0.05 was considered to be statistically significant. Sequence data is available at the European Nucleotide Archive (Accession number: PRJEB14074).

## Results

### Patient demographics & sampling

**Fig 1:**
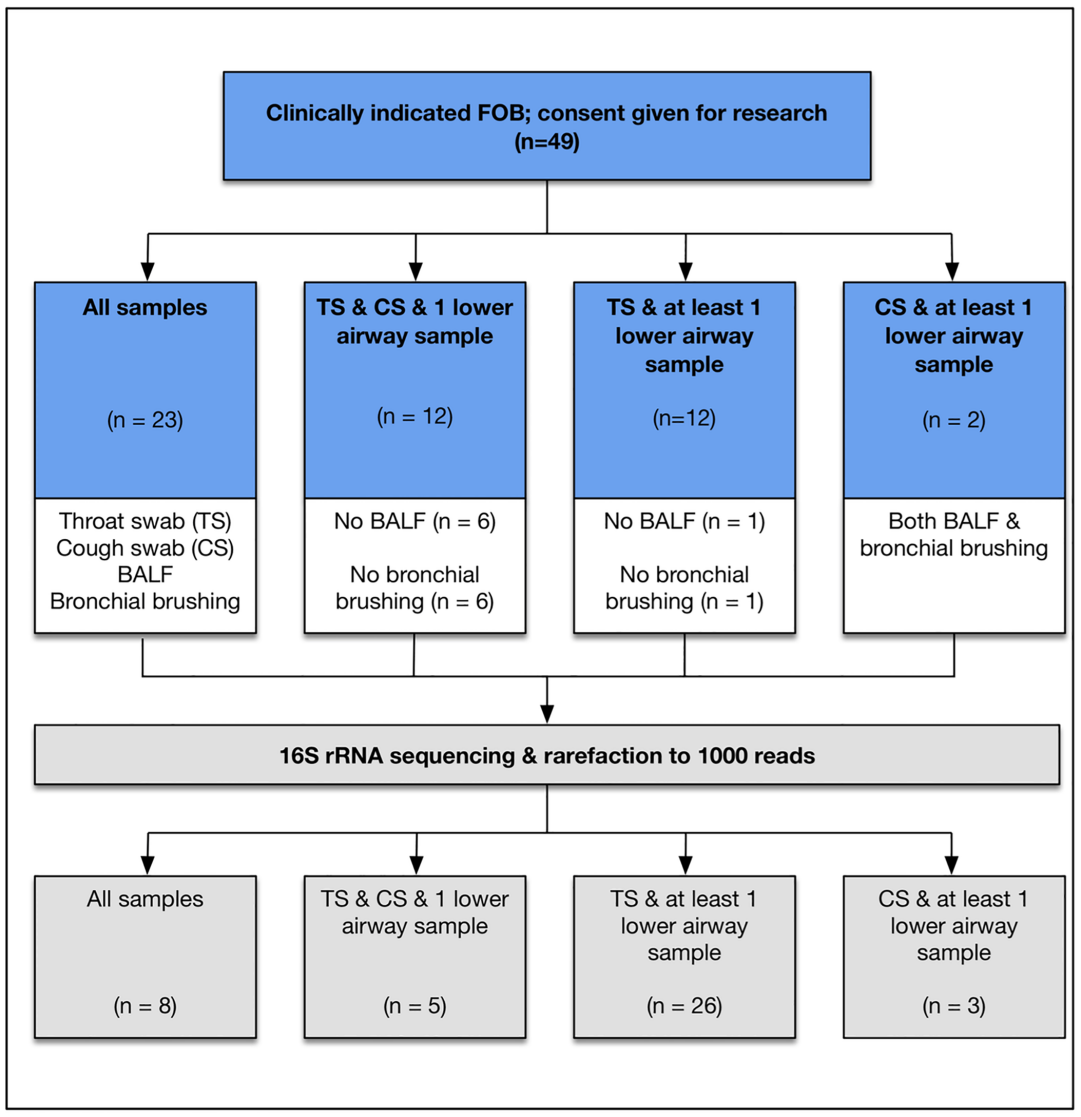
Illustration of the combinations of upper and lower airway samples taken. Forty-nine patients were recruited and in forty-seven patients, a throat swab and at least 1 lower airway sample was obtained for comparison. Samples for forty-two patients remained after rarefying to 1000 reads: 8 patients remained in whom all 4 samples were available; 39 had TS and at least a paired lower airway sample (either BALF, bronchial brushing or both), and 17 had CS and a paired lower airway sample. n—number of subjects. After rarefaction, some subjects had fewer samples for comparison and therefore moved into a different category.

Patient characteristics are detailed in Table 1. In total 47 TS, 37 CS, 42 BALF samples and 42 bronchial brushings were collected, with 23 of the 49 patients providing all sample types and a median of 3 samples collected per patient. Reasons for missed samples included: infants who were too young to perform a CS (n = 12); children who were unable to tolerate TS sampling due to gagging and vomiting (n = 2); lack of parental consent for bronchial brushings (n = 7), and insufficient BALF remaining for research after aliquoting for clinical purposes (n = 7). For the remaining 26 patients, at least one pair of upper and lower airway samples was collected thereby allowing comparisons to be made (Fig 1). Twenty-six of the 42 BALFs obtained were culture positive.

**Table 1:** Summary of patient characteristics (n = 49) in the comparison of the upper and lower airway microbiota. CSLD—chronic suppurative lung disease; CF—cystic fibrosis; NBS—newborn screened; PCD—Primary ciliary dyskinesia.

Demographic	Median (range) or n (%)
**Age** (years)	5.4 (0.1–16.2)
**Gender** (female)	29 (59)
**Underlying pathology**	
• CF (Newborn screened [NBS] infants)	• 12 (25)
• CF (not NBS)	• 5 (10)
• PCD	• 5 (10)
• Recurrent LRTI	• 24 (49)
• Upper airway pathology	• 2 (4)
• Haemoptysis	• 1 (2)
**Current symptoms**	
• Lower respiratory	• 10 (20)
• Upper respiratory	• 21 (42)
**Antibiotic usage**	
Current treatment course	14 (29)
• Intravenous (IV)	• 3 (6)
• Oral	• 8 (16)
• Nebulised	• 6 (12)
Oral prophylaxis	20 (41)
• Augmentin	• 2 (4)
• Azithromycin	• 9 (18)
• Flucloxacillin	• 9 (18)
**Inhaled corticosteroids**	12 (25)
**Bronchoscopy route**	
• Oral (Via laryngeal mask airway)	• 11 (22)
• Nasal	• 38 (78)

Indications for bronchoscopy included: chronic suppurative lung disease (CSLD—namely CF and Primary Ciliary Dyskinesia [PCD]) (45%) diagnosed on standard criteria[18], [19], and non-CSLD controls (55%). The majority of the controls (24/27, 89%) were being investigated for recurrent lower respiratory tract infections (LRTI). For those with CSLD, twenty (25%) were infants with CF diagnosed on newborn screening (NBS) undergoing a routine bronchoscopy at 3–5 months of age[20]. The age distribution of patients was skewed with a median of 5.4 years (0.1–16.2 years). Thirty-nine patients (80%) were clinically stable at the time of bronchoscopy. Eighteen patients (37%) had received a treatment course of antibiotics in the previous 30 days, with 29% receiving antibiotics at the time of bronchoscopy (either intravenous [IV], oral, nebulised or a combination).

### Sequencing

16S rRNA gene sequencing was performed on 168 extracted DNA samples. After initial processing and quality control, a total of 5.97 million reads were obtained with an average of 31,117 reads per sample (range 118–192,812 reads). Technical and PCR negative controls were examined to identify potential contaminant OTUs. Three individual OTUs were found to be highly abundant in technical and PCR control samples and were removed: *Burkholderia* (OTU ID 1606), *Undibacterium* (OTU ID 1727) and *Ralstonia* (OTU ID 1703). In addition, the genera *Bradyrhizobium* spp., *Sediminibacterium* spp. and *Methylobacterium* spp. were removed as these have previously been identified as common reagent contaminants[21]. A small significant batch effect was found between the two sequencing runs using Bray Curtis dissimilarity (r^2^ = 0.03, *P* = 0.001) and unweighted UniFrac scores (r^2^ = 0.01, *P* = 0.03), but not the weighted UniFrac score (r^2^ = 0.01, *P* = 0.69).

Prior to downstream processing, data was filtered to remove OTUs with less than 20 total reads. Samples were rarefied to 1,000 reads and any sample with fewer than 1,000 reads removed (rationale for rarefaction level chosen detailed in the S1 Appendix and S3 Fig). After rarefaction 34/42 (81%) of the BALFs, 36/42 (86%) bronchial brushings, 44/47 (94%) TS and 17/37 (46%) CS remained. CS sequenced poorly, mostly likely due to having a very low biomass, and consequently post rarefaction only 8 patients remained in whom all 4 samples (TS, CS, BALF and bronchial brushing) were available for comparison. In contrast, post rarefaction 39 patients had a TS and at least a paired lower airway sample remaining with 17 having a CS and paired lower airway sample (Fig 1).

### Comparison of the upper and lower airway microbiota

**Fig 2:**
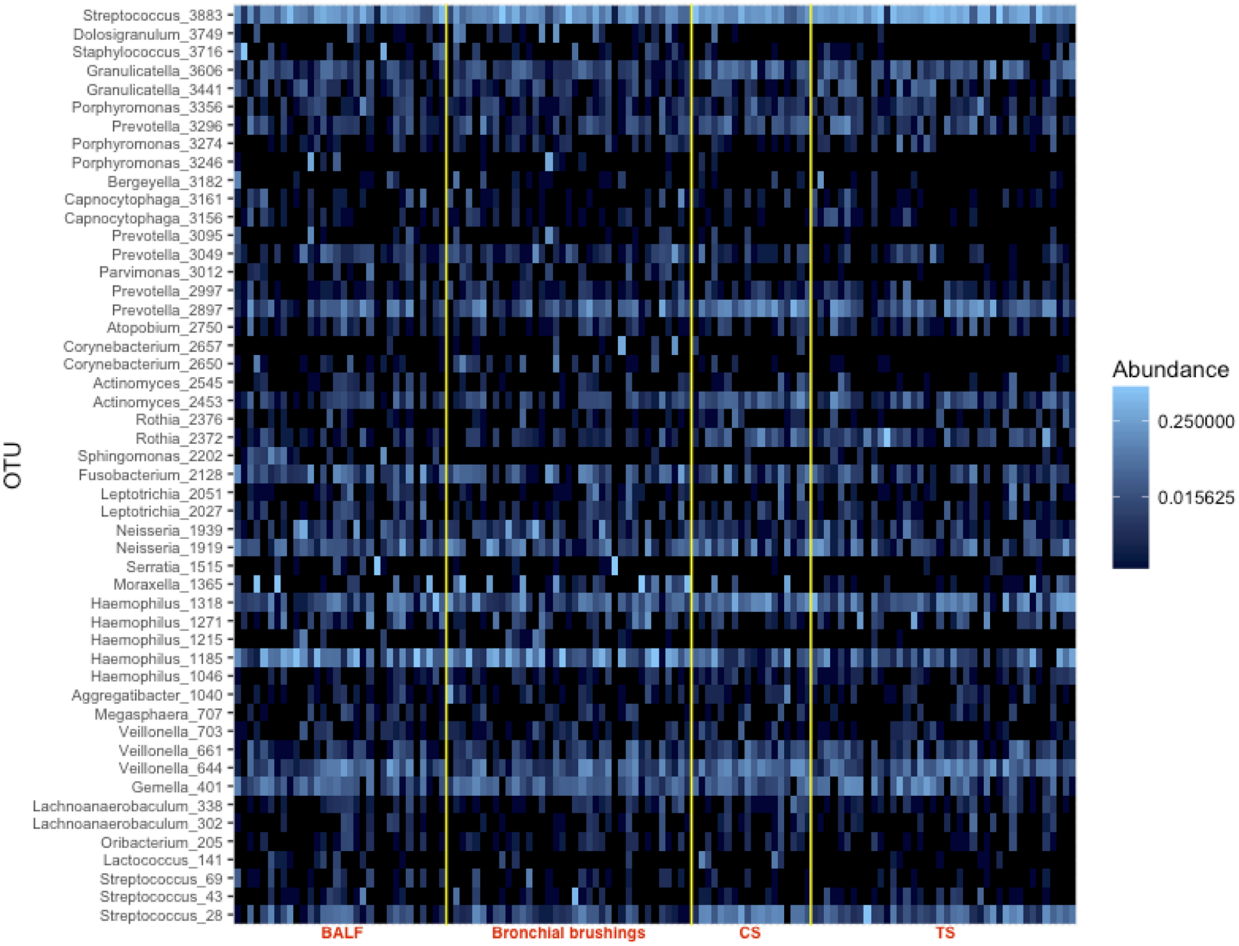
Heatmap showing similarities in the relative abundances of the top 50 OTUs present between upper (throat swabs [TS] and cough swabs [CS] and lower airway samples (bronchoalveolar lavage fluid [BALF] and bronchial brushings).

For BALF and bronchial brushings, the three most common genera were identical: *Haemophilus* spp. (23.6% and 24.6% of total reads respectively), *Streptococcus* spp. (20.3% and 20.5%) and *Prevotella* spp. (6.5% and 7.9%). Similar genera were seen in TS but with *Streptococcus* spp. the most common (39.5%) followed by *Haemophilus* spp. (15.4%) and *Prevotella* spp. (8.7%). At an OTU level, there was no difference observed between upper and lower airway samples (Fig 2).

Comparing alpha diversity between paired BALF and bronchial brushings (N = 26), no significant differences were found in richness (median for BALF = 57.5 (range 12–87), for bronchial brushings = 50, (range 14–101), *P* = 0.578), evenness (median for BALF = 0.60 [range 0.16–0.75], for bronchial brushings = 0.55 [range 0.13–0.70] *P* = 0.084), or by the Shannon Diversity Index (median for BALF 2.46 [range 0.39–3.22], for bronchial brushings 2.18 [range 0.37–3.19], *P* = 0.173). Similarly no differences were observed for beta diversity (Bray Curtis dissimilarity index [*P* = 0.650, r^2^ = 0.005], unweighted UniFrac score [*P* = 0.114, r^2^ = 0.02], weighted UniFrac score [*P* = 0.255, r^2^ = 0.008]). Consequently where both BALF and bronchial brushings were available for comparison with TS, bronchial brushings were used.

**Fig 3:**
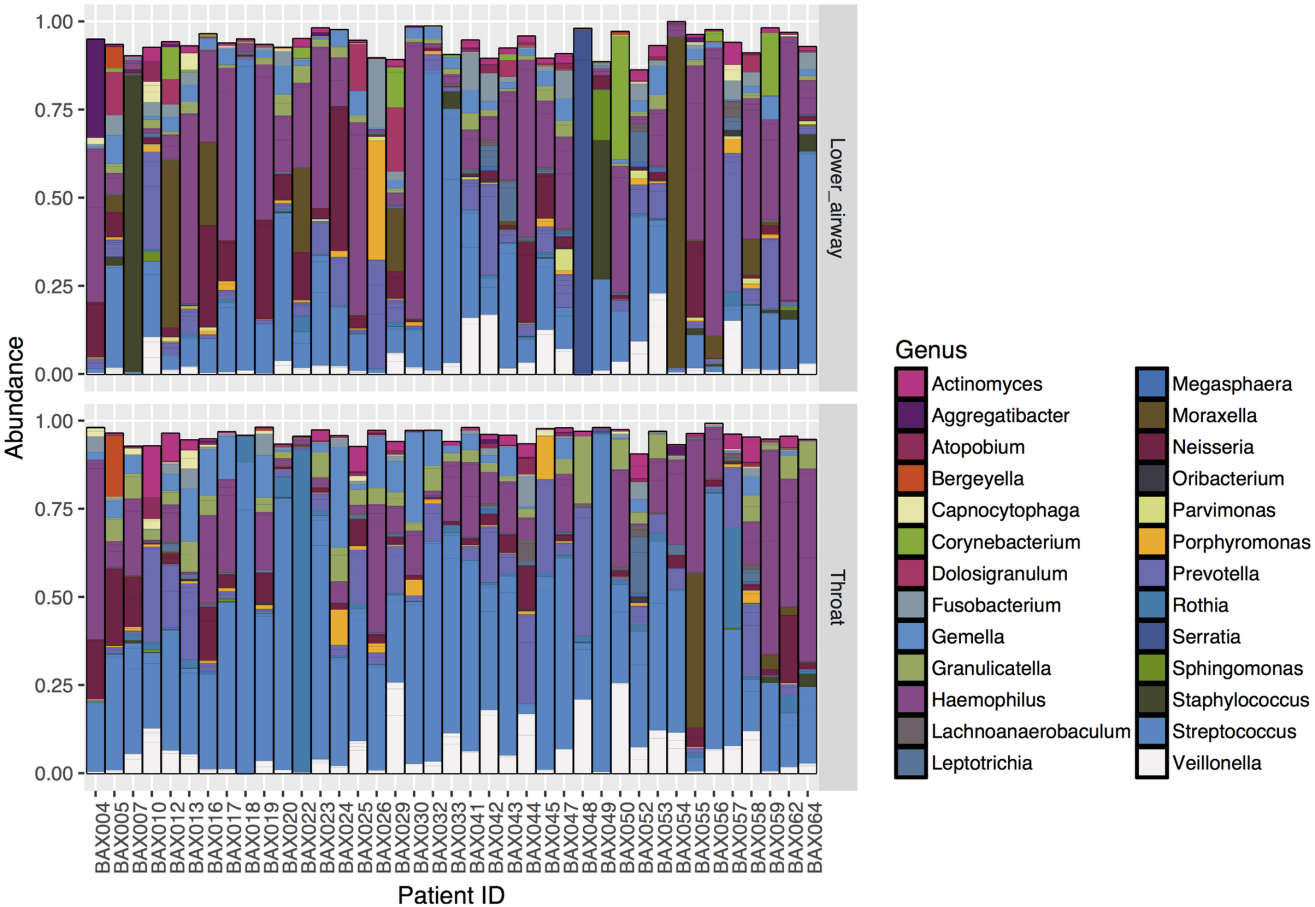
Individual patient barplots (N = 39) comparing paired lower airway samples and TS for the 50 most common OTUs. The relative abundance of OTUs for each sample is shown with TS below and their corresponding lower airway sample above. Bars are of uneven heights due to the presence of low abundance “other” OTUs which have not been included in the plot. This illustrates that the airway microbiota is highly individual with variable degrees of similarity between TS and lower airway samples.

Looking at individual patient barplots the airway microbiota was revealed to be highly individual (S4 Fig), with some similarities observed between TS and lower airway samples (Fig 3). *Streptococcus* spp., however, appears to be more abundant in TS compared to lower airway samples. Communities with low evenness were in most cases dominated by a single organism. There was a trend for the dominant organism in the lower airway sample to be the same bacterium isolated by routine clinical culture of BALF (S1 Appendix and S4 Fig).

**Fig 4:**
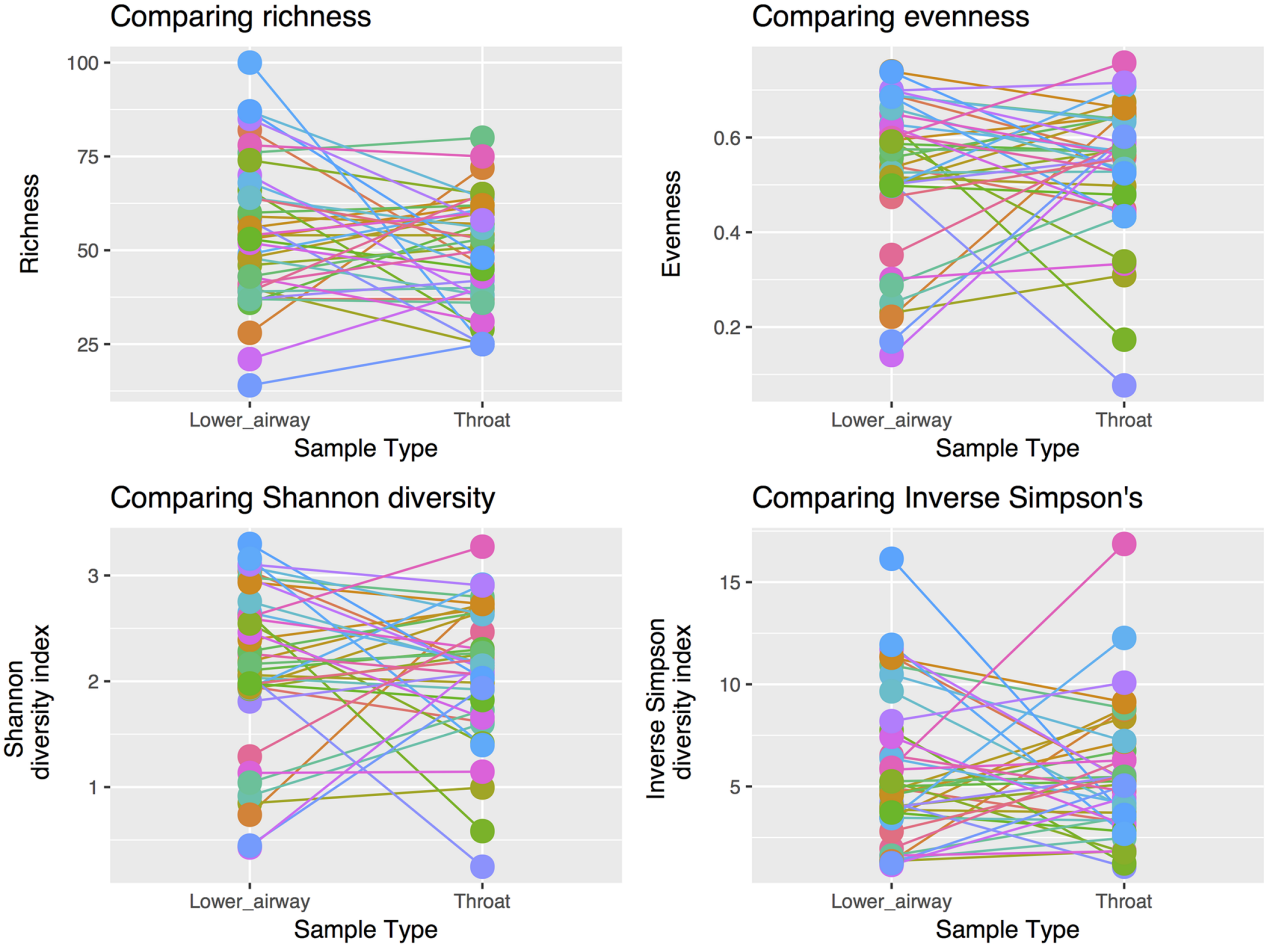
Alpha-diversity comparisons between lower airway samples and throat swabs. No significant difference was seen in: (a) richness (t_(38)_ = 1.6523, *P* = 0.107); (b) evenness (W_(38)_ = 367, *P* = 0.756), and (c) Shannon diversity index (W_(36)_ = 384, *P* = 0.940).

Comparing TS and either BALF or bronchial brushings (N = 39), no significant difference was found in richness (median for lower airway samples = 51 [range 14–101], for TS = 47 [range 25–70], *P* = 0.120), evenness (median for lower airway samples = 0.55 [range 0.13–0.76], for TS = 0.56 [range 0.09–0.75], *P* = 0.809) or by the Shannon Diversity Index (median for lower airway samples = 2.18 [range 0.37–3.29], for TS = 2.13 [range 0.31–3.17], *P* = 0.777, Fig 4).

**Fig 5:**
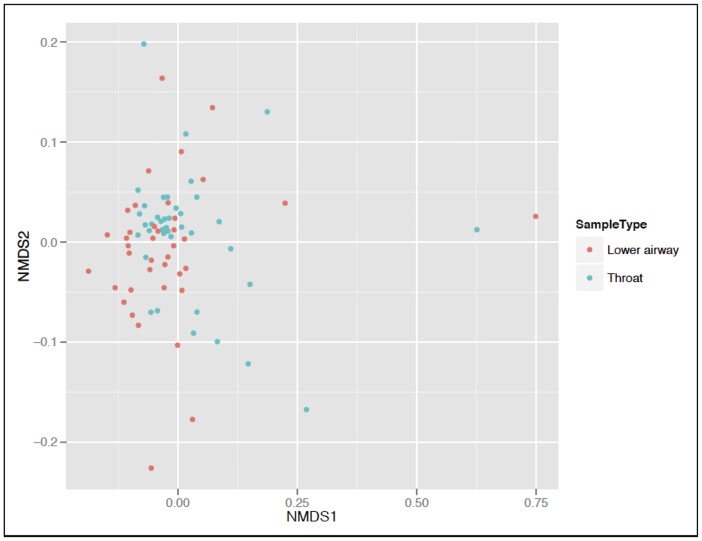
Non-metric multidimensional scaling (NMDS) plot comparing the UniFrac score between patients. This shows similarity in the clustering pattern between lower airway samples and throat swabs.

For those individuals showing a difference in alpha diversity between upper and lower airway samples, Bland-Altman plots of alpha diversity measurements revealed somewhat less agreement between TS and lower airway samples with low evenness (S5 Fig). This again suggests that there is less concurrence between TS and lower airway samples in the presence of a dominant organism. The mean Shannon Diversity Index was 2.09 (standard deviation [SD 0.64] for TS and 2.12 [SD 0.78] for lower airway samples). A small significant difference (*P* = 0.001) was found in beta-diversity between TS and lower airway samples using the Bray Curtis, unweighted and weighted UniFrac scores (r^2^ = 0.06, 0.03 and 0.06 respectively [Fig 5]).

Considering genera, 80% (72 out of 90) were in common between lower airway samples and TS, whilst 15.6% (14 out of 90) were unique to BALF and 4.4% (4 out of 90) were unique to TS. None of the genera unique to BALF were grown on bacterial culture of the same sample. The few genera different between upper and lower airway samples were present in very low relative abundance (< 0.1%).

Spearman’s rank correlation testing showed good correlation between the relative abundances of genera and OTUs present when comparing lower airway samples with TS (genera level: r = 0.776, *P* < 0.001; OTU level: r = 0.557, *P* < 0.001). Using multiple paired t-tests with Benjamini-Hochberg correction to compare the relative abundance of genera, a significantly higher relative abundance of *Streptococcus* spp. was seen (t = -182, *P* < 0.0001, *P*_adj_ = 0.0009) for TS. No other genus was significantly different between TS and lower airway samples (*P*_adj_ > 0.05) (Table 2).

**Table 2:** Comparing the mean relative abundance (percent) of the most common or clinically important genera between TS and paired lower airway samples (BALF or bronchial brushing). Using multiple paired t-tests with Benjamini-Hochberg correction, only *Streptococcus* spp. was significantly different (*P*_**(adj)**_ = 0.0009). TS—throat swab; NS—non-significant (*P*_**(adj)**_ > 0.05). SD—standard deviation.

Genus	Lower airway mean (SD)	TS mean (SD)	*P*_(adj)_-value
***Haemophilus* spp.**	25.2 (24.4)	15.6 (16.2)	0.35
***Streptococcus* spp.**	21.5 (22.2)	39.7 (22.8)	**0.0009**
***Prevotella* spp.**	7.7 (9.9)	8.3 (10.0)	0.89
***Neisseria* spp.**	6.9 (10.1)	4.6 (6.3)	0.47
***Moraxella* spp.**	6.2 (17.4)	1.4 (7.0)	0.47
***Veillonella* spp.**	4.2 (5.6)	6.8 (7.1)	0.35
***Staphylococcus* spp.**	4.1 (16.3)	0.2 (0.7)	0.47
***Gemella* spp.**	3.0 (2.6)	5.3 (7.3)	0.42
***Fusobacterium* spp.**	2.6 (3.8)	1.3 (2.0)	0.42
***Serratia* spp.**	2.6 (15.9)	0.05 (0.1)	0.55
***Corynebacterium* spp.**	2.2 (6.6)	0.03 (0.1)	0.35
***Granulicatella* spp.**	1.7 (1.7)	4.2 (3.9)	0.05
***Dolosingranulum* spp.**	1.6 (4.2)	0.03 (0.1)	0.35
***Porphyromonas* spp.**	1.6 (5.4)	1.2 (2.5)	0.79
***Leptotrichia* spp.**	1.1 (2.7)	1.0 (2.6)	0.83
***Rothia* spp.**	0.5 (1.0)	4.5 (15.6)	0.42
***Burkholderia* spp.**	0.1 (0.1)	0.00 (0.02)	0.35
***Pseudomonas* spp.**	0.04 (0.1)	0.04 (0.1)	0.90
***Ralstonia* spp.**	0.04 (0.1)	0.01 (0.03)	0.42

Although CS sequenced poorly, when successfully sequenced they showed similarities in diversity with lower airway samples (see S1 Appendix).

### Clinical variables influencing correlation between the upper and lower airway microbiota

To determine whether specific clinical features underpinned the differences in similarly between upper and lower airway samples (Fig 3), beta-diversity testing was performed using a PERMANOVA including a total of 11 variables—those listed in Table 1 as well as “Patient ID” (i.e. which patient was sampled) and “Sample type”. The influence of FOB route and underlying pathology were tested in separate PERMANOVAs using FOB samples or TS only respectively. Two variables were found to exert a large influence on community structure. “Patient ID” had the largest influence accounting for 47.0–53.8% of the variance (for the unweighted UniFrac and Bray Curtis dissimilarity scores respectively, *P* = 0.001), whilst the underlying pathology accounted for 18.7%– 22.5% (unweighted UniFrac and Bray Curtis dissimilarity scores respectively, *P* ≤ 0.006) (Table 3). FOB route accounted for 5.43%–8.16% of the variance (unweighted and weighted UniFrac respectively, *P* < 0.05), with fewer *Corynebacterium* spp. (OTU 2657), *Moraxella* spp. (OTU 1365) and *Dolosingranulum* spp. (OTU 349) present in FOB performed nasally as opposed to FOB through the oral route. Other significant variables, but similarly of more minor influence, included the use of prophylactic antibiotics and nebulized antibiotics. Patient age was not a significant influence on community structure.

**Table 3:** Beta diversity summaries of significant clinical variables influencing community structure. Adonis (PERMANOVA) results shown are for those variables which were statistically significant (*P* < 0.05) using the Bray Curtis dissimilarity score. IV—intravenous. FOB—fibreoptic bronchoscopy.

Variable & diversity measurement	r^2^	*P*-value
**Sample Type**	0.06	0.001
**Patient ID**	0.54	0.001
**Underlying pathology**	0.20	0.001
**Lower respiratory tract symptoms**	0.02	0.003
**Nebulised antibiotics**	0.02	0.003
**Current IV antibiotics**	0.01	0.012
**FOB route**	0.08	0.001

## Discussion

In summary, the present study has revealed that TS sequence well (94% of samples sequenced) in contrast to CS that sequenced poorly (44%). We attribute this to the limited biomass and recoverable microbial DNA for CS and the use of CS as a sample for sequencing is therefore not recommended.

When comparing TS with lower airway samples (either BALF or bronchial brushings), a strong correlation was seen in the relative abundance of genera and there was no significant difference in within sample alpha diversity. Whilst a significant difference in beta diversity was seen between sample types, the degree of variation due to sample site and other clinical variables, such as antibiotic usage, was small. This was consistent when testing whether similar organisms were present between samples (Bray Curtis dissimilarity) and when testing the relative abundance and phylogenetic relationships between samples (UniFrac and weighted UniFrac scores).

There was greater variability between disease states (up to 22.5%) than between upper and lower airway samples. However, the number of children with discordant lower and upper airway samples precludes the use of TS for clinical decision making in individuals. Nevertheless TS contain valuable research information that could be used to explore the development of the lower airway microbiota in groups of children with different diseases. For example, TS could be used to determine whether the more benign clinical course of PCD compared to that of CF relates to different temporal evolution of the lower airway microbiota.

Discordant dominance of an OTU in either the upper or lower airway sample led to greater dissimilarity between TS and lower airway samples. For those samples showing the greatest dissimilarity, there was a trend for the dominant organism in BALF to be the same organism grown on bacterial cultures of the same fluid. This could suggest overgrowth of an organism in an individual sample, or low biomass in that sample allowing artificial dominance of an organism. Quantitative PCR was not performed as part of this study and may have helped determine whether low biomass contributed to this observation. Therefore, where samples are dominated by an individual genus, the results should be treated with caution and repeat upper airway sampling paired with culture dependent microbiology is wise. Upper airway samples may therefore fail to detect dominant pathogens in the lower airways and vice versa, limiting clinical use in an individual child but also a consideration for the design of research studies.

Two main factors influenced the degree of variation in the airway microbiota. The patient sampled had the greatest influence (53.8% of variation, Bray Curtis dissimilarity score). This confirms that the microbiota is individual to a patient[22–24]. The underlying pathology accounted for up to 22.5% of variation in TS, suggesting TS detect disease differences which may be useful in longitudinal studies of the airway microbiota when comparing different patient groups.

Our findings in 49 children are similar to those of Charlson *et al*.[13], who demonstrated similarity of the microbiota along the respiratory tree in 6 adults (including smokers), and Boutin *et al*.[10] who demonstrated similarities between TS and sputum samples in 20 adults and children with CF with a mean age of 16.1 years. Similarly, Marsh *et al*.[25] compared upper airway samples (nasopharyngeal and oropharyngeal swabs) with BALF sampled from a single lobe and found upper airway samples were a reliable surrogate in 69% of children with either idiopathic bronchiectasis, protracted bacterial bronchitis or healthy airways. Our current study we believe is the first to compare the microbiota using both TS and CS and lower airways samples in young children with CSLD (CF or PCD) and non-CSLD controls (mainly recurrent LRTI), which is representative of the pathologies frequently encountered in Paediatric Respiratory Medicine. This is important since children are less able to spontaneously expectorate and in whom finding a reliable surrogate for lower airway sampling which can be obtained frequently is particularly pertinent.

Our study may be underpowered as we were unable to perform an *a priori* power calculation (S1 Appendix). A significant difference in beta-diversity was however observed meaning differences between groups of patient samples could be detected. Nonetheless caution should be taken in relation to the interpretation of the CS data given the low sample size (N = 17) and their very low biomass, rendering them at increased risk of contamination by spurious OTUs. Low biomass also will have contributed to the variation in sequencing depth between samples and the need to rarefy to a level balancing capturing OTUs and retaining sufficient samples for paired comparisons. However, higher sequencing depths do not lead to improved identification of ecological patterns[26].

Eighteen patients (37%) had received a course of antibiotics in the previous 30 days and 41% were prescribed prophylactic antibiotics. Whilst this could potentially introduce a bias in our results by influencing both the upper and lower airway microbiota so that they show greater similarity, it would not have been ethically permissible to stop a clinically indicated treatment for the purposes of this study, and indeed many of these patients are prescribed antibiotics in routine clinical practice. Nonetheless, we accept that extrapolation of our results to antibiotic-naïve children should be cautious.

Ideally, all bronchoscopies would have been performed either via an LMA or endotracheal tube in order to minimize risk of contamination of the bronchoscope. The route was determined by clinical considerations. Bronchoscopy route accounted for up to 8.16% variance in community structure, much less than the variance due to the individual patient (53.8%) or the underlying disease (22.5%). Greater abundance of organisms associated with the nasal passages such as *Corynebacterium* was seen in samples where bronchoscopy was performed transnasally. Similarly, contamination of upper airway samples with the oral microbiota cannot be excluded, although care was taken to limit for this by avoiding contact with the oral cavity during sampling and not using suction until the bronchoscope was below the vocal cords. Nasopharyngeal samples were not collected and have previously been found to be highly diverse in young children[27].

BALF was only collected and pooled from two lung lobes in this study. As lobar differences exist in bacterial distribution[28], ideally all six lobes would have been sampled to determine geographical consistency More work is needed on intra-lobar differences and the relationship with upper airway cultures.

A small significant batch effect was seen between sample plates using the Bray Curtis and weighted UniFrac scores (r2 = 3.32% and 3.82% respectively). Except for 10 samples, all samples from an individual patient were however, run on the same plate thereby limiting the impact of any batch effect.

In summary, CS sequenced poorly and their use cannot be recommended for non-culture based microbiota studies. Considering TS as a surrogate for lower airway samples, although representative at a community level and at this level can demonstrate disease differences, throat swabs can show substantial differences at the individual patient level. Notably larger differences are observed when samples are dominated by an individual organism, the latter being identified by both molecular and culture-dependent techniques. A combination of these two techniques may be an important consideration and advantageous in order to obtain a comprehensive assessment of a patient’s airway bacterial community. Consequently TS do not have utility for individual clinical decision making but they provide an opportunity as a research tool for tracking longitudinal changes in groups of patients with, for example, CF and PCD.

## Supporting information

S1 Appendix Supplementary methods and results for the comparison of the upper and lower airway microbiota in children with chronic lung diseases. (DOCX)

S1 Fig Illustration of the methodological steps in sample processing from DNA extraction to 16S rRNA gene sequencing using the Illumina MiSeq. (TIF)

S2 Fig Diagram illustrating the analysis pipeline for sequences obtained from the Illumina MiSeq. Upstream analyses were performed in QIIME and downstream analyses were performed in Phyloseq in R.
(TIF)

S3 Fig Rarefaction curves with yellow lines denoting the number of OTUs sampled at 1,000 reads and 3,000 reads. This illustrates that an asymptote is reached by 1,000 reads. At this threshold, the majority of OTUs have been sampled and little additional information is obtained at higher rarefaction levels. Consequently a rarefaction level of 1,000 reads was chosen.
(TIF)

S4 Fig Illustrating individual patient barplots (N = 40) organised from those showing the greatest similarity between upper and lower airway samples to those showing the least similarity (determined by Bray Curtis dissimilarity). Only BALF samples were sent for bacterial culture as part of routine clinical care. The results of BALF culture and disease group are also detailed.
(PDF)

S5 Fig Bland Altman plots showing agreement between TS and lower airway samples in alpha diversity measurements illustrated by (a) richness, (b) evenness and (c) Shannon Diversity Index. Overall agreement is seen between samples, apart from at low levels of evenness and Shannon Diversity.
(TIF)

S1 Table Summary of sequencing quality statistics. (DOCX)
