# Supplementary material for: Comparison of the upper and lower airway microbiota in children with chronic lung diseases
Source: PLoS One. 2018 Aug 2;13(8):e0201156. doi: 10.1371/journal.pone.0201156 (PMC6071972; doi:10.1371/journal.pone.0201156)
Supplement: S1 Appendix — (DOCX) [file pone.0201156.s001.docx]

S1 Appendix.

**Supplementary Methods and Results for the Comparison of the Upper and Lower Airway Microbiota in Children with Chronic Lung Diseases**

# Methods

### Subjects and sampling

Swabs were collected using a sterile, cotton-tipped swab (Sterilin, UK). Cough swabs (CS) were collected by placing a swab as far to the back of the child’s throat as possible and asking the child to give two strong coughs, taking care not to touch any surface in the oropharynx. Throat swabs (TS) were taken by rubbing a swab back and forth 5 times in front of the uvula, whilst taking care not to touch any other oropharyngeal surface. [[S1](#_ENREF_1)],[[S2](#_ENREF_2)]. A tongue depressor was used for both TS and CS to limit contamination from the tongue and oral mucosa. Where both CS and TS were collected, TS were collected first and then CS. Fibreoptic bronchoscopy (FOB) was performed either nasally or via a laryngeal mask airway (LMA) depending on the clinical context of the bronchoscopy and the preference of the anaesthetist[[S3](#_ENREF_3)]. Suction was not used until the tip of the bronchoscope had passed beyond the vocal cords in order to minimize contamination from the upper airways. Bronchoalveolar lavage fluid (BALF) was collected in accordance with the European Respiratory Society Task Force recommendations[[S4](#_ENREF_4)]. To account for lobar differences, 2 lobes were sampled and pooled. Bronchial brushings were collected using either a 1.2mm diameter unsheathed or 2mm sheathed bronchial cytology brush (Olympus Keymed, UK) depending on patient age and size. The order of sample collection was consistent between all patients and was: TS then CS then BALF and finally bronchial brushings. TS and CS were collected in the 2 hour period prior to FOB with the patient awake to mimic the clinical settings in which swabs are commonly collected. Technical controls were taken to test for contamination using blank swabs in the case of TS and CS whilst for BALF and brushings sterile 0.9% saline or blank bronchial brushings were passed down the bronchoscope prior to performing FOB. All samples were placed immediately onto dry ice and transferred to -80^o^C where they were stored until DNA extraction. Due to a lack of pilot data, an *a priori* power calculation could not be performed; sample size was opportunistic.

**16S rRNA amplicon library preparation and sequencing**

### With the exception of 10 samples, all samples from an individual patient were run on the same plate, limiting the impact of any batch effect on comparisons of upper and lower airway samples. The ten exceptions were: TS for patient subject numbers 16, 44, 48 and 49; CS for subject number 18; BALF for subject numbers 24, 47, 55, and bronchial brushings for subject numbers 34 and 45.

### Data analysis.

**Sequence analysis in QIIME.**

Upstream analyses were performed using QIIME (version 1.9.0)[[S5](#_ENREF_5)]. First the 8 base pair (bp) forward and reverse barcodes were combined into a single 16 bp unique identifier. Three prime ends of the sequences were quality trimmed and read through adaptors were removed using Trim Galore. Forward and reverse reads were then combined into a single read using fastq-join[[S6](#_ENREF_6)] with a requirement of a minimum overlap length of 200 bp with ≤ 10% misalignment. For any instances of mismatch, the base with the highest quality score was retained.

Next samples were de-multiplexed in order to assign sequences back to their original sample. Quality filtering was next performed with Phred scores assessed for each base and any read with an average score below the minimum quality threshold (< Q30) being removed. If 10 consecutive bases failed to meet the threshold, the read was truncated at this point. The resulting sequence was discarded if < 70% of consecutive bases in the combined read were ≥ Q30. During de-multiplexing, multiple sequencing runs containing samples from the same study group were combined into one dataset. Sequences generated from PhiX control library were removed after de-multiplexing by aligning surviving reads against the PhiX genome using the Burrows-Wheeler Alignment tool[[S7](#_ENREF_7)].

Next, taking the resulting sequences, operational taxonomic units (OTUs) were assigned using an open reference approach with sequences clustered against the SILVA ribosomal RNA gene database (Version 115)[[S8](#_ENREF_8)]. OTU picking was performed in UCLUST (Version 1.2.22q) [[S9](#_ENREF_9)] with a threshold of 97% sequence similarity, corresponding to a species level.

The most abundant sequence was picked as a representative for the OTU cluster. Sequences were aligned using the Python Nearest Alignment Space Termination (PyNAST) tool [[S10](#_ENREF_10)] and a lanemask filtered alignment of the SILVA database [[S8](#_ENREF_8)]. Removal of chimeric sequences was performed using ChimeraSlayer [[S11](#_ENREF_11)] and FastTree (Version 2.1.3) [[S12](#_ENREF_12)] was used to construction of a phylogenetic tree. Species identity of the representative sequence was then assigned using UCLUST (Version 1.2.22q) [[S9](#_ENREF_9)] and the SILVA database [S[8](#_ENREF_8)]. An OTU table was constructed detailing the OTUs present per sample. The OTU table, phylogenetic tree and representative sequences were uploaded into R for downstream analyses (performed in Phyloseq).

Rarefaction was performed to control for the variability in sequencing depth between samples. The rarefaction level was chosen by examining rarefaction curves to determine the number of reads at which most samples reached an asymptote (Figure E3) and a rarefaction level of 1000 reads was chosen.

**Results**

**Comparison of upper and lower airway microbiota**

As there is no standardised Bray Curtis dissimilarity threshold at which samples are considered to be highly dissimilar, from examining individual patient barplots, greater dissimilarity was seen between TS and lower airway samples with a score of ≥ 0.7. There were twelve patients with Bray Curtis scores above this threshold. Of these, five had positive growths on culture of BALF specimens (excluding growths of “upper respiratory tract flora only”, n = 3). Organisms cultured were *Staphylococcus aureus* (n = 2), *Haemophilus influenzae* (n = 2) and *Serratia marcescens* (n = 1); the dominant genus in their corresponding sequenced sample was identical, namely *Staphylococcus* spp.; *Haemophilus* spp. and *Serratia* spp*.* respectively. Four patients had no growth on culture of BALF, of which three had a dominant organism on sequencing (*Streptococcus* spp., *Neisseria* spp. and *Moraxella* spp.).

**Comparison of cough swabs with lower airway samples**

Although CS sequenced poorly, when successfully sequenced they showed some similarities in relation to diversity with their paired lower airway samples. The three most common genera in CS were similar to those seen in BALF and bronchial brushings with *Streptococcus* spp. the most common (29.6% of reads), followed by *Haemophilus* spp. (13.6%) and *Prevotella* spp. (13.5%).

Comparing CS with their respective lower airway samples (n=17), no significant difference was seen in richness (t_(16)_ = -0.831, *P* = 0.418), evenness (W = 40, *P* = 0.089) or the Shannon diversity index (W = 48, *P* = 0.190). Similarly a significant difference was seen in the Bray Curtis (r^2^ = 0.08, *P* = 0.002) and weighted UniFrac scores (r^2^ = 0.06, *P* = 0.029), but not in the unweighted UniFrac score (r^2^ = 0.03, *P* = 0.2). Considering genera, 70.4% (57 out of 81) were common to both lower airway samples and CS whilst 19.6% (16 out of 81) were unique to lower airway samples and 10.0% (8 out of 81) were unique to CS (Figure 3.15). Spearman’s rank correlation testing showed good correlation between the relative abundances of OTUs present when comparing lower airway samples with CS (genera level: rho = 0.795, *P* < 0.001; species level: rho = 0.579, *P* < 0.001).

**References:**

S1. Ahmed B, Bush A, Davies JC (2014) How to use: bacterial cultures in diagnosing lower respiratory tract infections in cystic fibrosis. Arch Dis Child Educ Pract Ed 99: 181-187.

S2. Cardenas PA, Cooper PJ, Cox MJ, Chico M, Arias C, et al. (2012) Upper airways microbiota in antibiotic-naive wheezing and healthy infants from the tropics of rural Ecuador. PLoS One 7: e46803.

S3. Midulla F, de Blic J, Barbato A, Bush A, Eber E, et al. (2003) Flexible endoscopy of paediatric airways. Eur Respir J 22: 698-708.

S4. de Blic J, Midulla F, Barbato A, Clement A, Dab I, et al. (2000) Bronchoalveolar lavage in children. ERS Task Force on bronchoalveolar lavage in children. European Respiratory Society. Eur Respir J 15: 217-231.

S5. Caporaso JG, Kuczynski J, Stombaugh J, Bittinger K, Bushman FD, et al. (2010) QIIME allows analysis of high-throughput community sequencing data. Nat Methods 7: 335-336.

S6. Aronesty E (2011) ea-utils: Command-line tools for processing biological sequencing data. Expression Analysis, Durham, NC.

S7. Li H, Durbin R (2010) Fast and accurate long-read alignment with Burrows-Wheeler transform. Bioinformatics 26: 589-595.

S8. Quast C, Pruesse E, Yilmaz P, Gerken J, Schweer T, et al. (2013) The SILVA ribosomal RNA gene database project: improved data processing and web-based tools. Nucleic Acids Research 41: D590-D596.

S9. Edgar RC (2010) Search and clustering orders of magnitude faster than BLAST. Bioinformatics 26: 2460-2461.

S10. Caporaso JG, Bittinger K, Bushman FD, DeSantis TZ, Andersen GL, et al. (2010) PyNAST: a flexible tool for aligning sequences to a template alignment. Bioinformatics 26: 266-267.

S11. Haas BJ, Gevers D, Earl AM, Feldgarden M, Ward DV, et al. (2011) Chimeric 16S rRNA sequence formation and detection in Sanger and 454-pyrosequenced PCR amplicons. Genome Res 21: 494-504.

S12. Price MN, Dehal PS, Arkin AP (2010) FastTree 2--approximately maximum-likelihood trees for large alignments. PLoS One 5: e9490.

**Figure legends**

**S1 Fig:** Illustration of the methodological steps in sample processing from DNA extraction to 16S rRNA gene sequencing using the Illumina MiSeq.

**S2 Fig:** Diagram illustrating the analysis pipeline for sequences obtained from the Illumina MiSeq. Upstream analyses were performed in QIIME and downstream analyses were performed in Phyloseq in R.

**S3 Fig:** Rarefaction curves with yellow lines denoting the number of OTUs sampled at 1000 reads and 3000 reads. This illustrates that an asymptote is reached by 1000 reads. At this threshold, the majority of OTUs have been sampled and little additional information is obtained at higher rarefaction levels. Consequently a rarefaction level of 1000 reads was chosen.

**S4 Fig:** Illustrating Individual Patient barplots (n = 40) organised from those showing the greatest similarity between upper and lower airway samples to those showing the least similarity (determined by Bray Curtis dissimilarity). Only BALF samples were sent for bacterial culture as part of routine clinical care. The results of BALF culture and disease group are also detailed.

**S5 Fig:** Bland Altman plots showing agreement between TS and lower airway samples in alpha diversity measurements illustrated by (a) richness, (b) evenness and (c) Shannon Diversity Index. Overall agreement is seen between samples, apart from at low levels of evenness and Shannon Diversity.
