# Supplementary figures and images for: Comparison of the upper and lower airway microbiota in children with chronic lung diseases
Source: PLoS One. 2018 Aug 2;13(8):e0201156. doi: 10.1371/journal.pone.0201156 (PMC6071972; doi:10.1371/journal.pone.0201156)

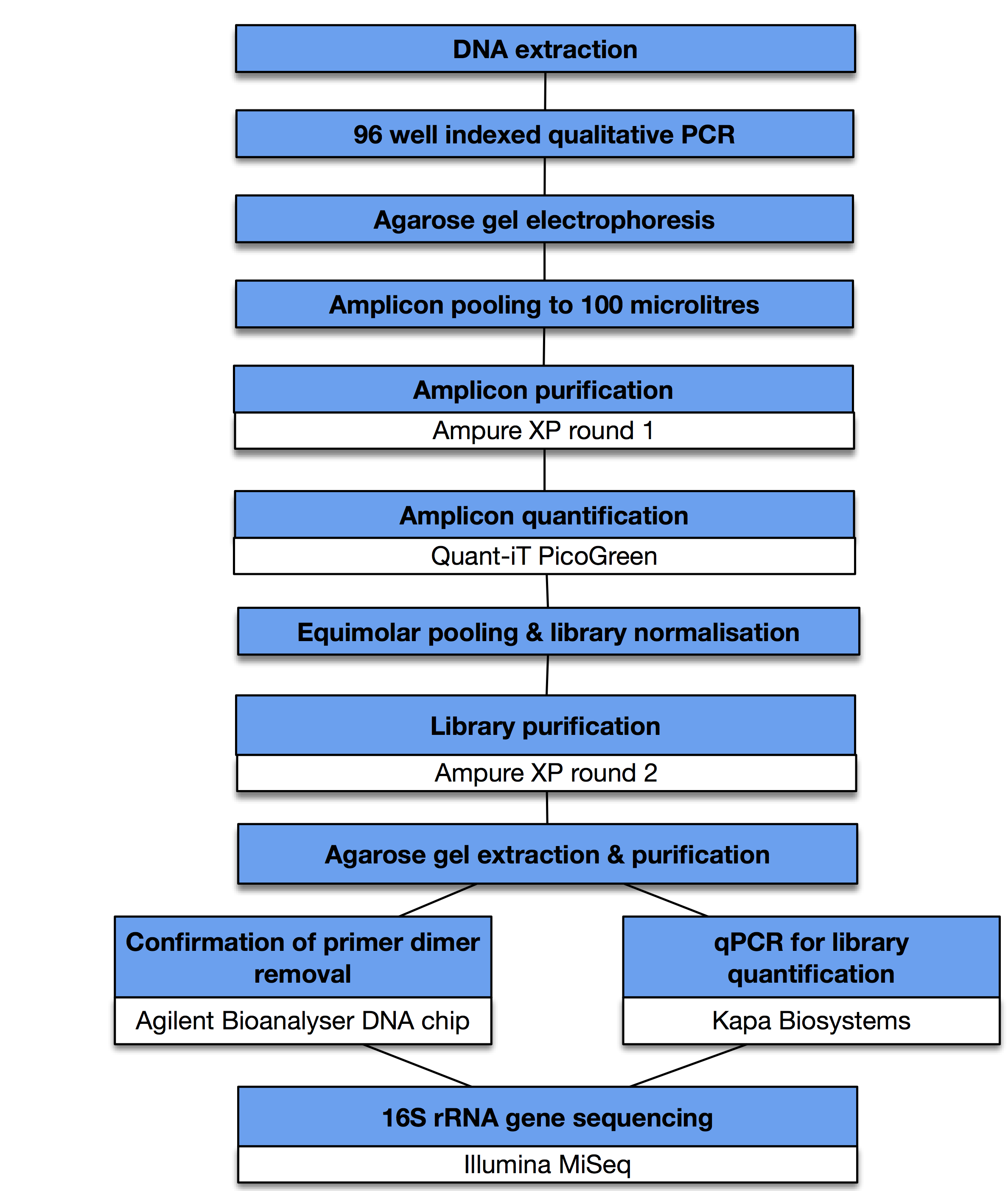

Supplement: S1 Fig — (TIF) [file pone.0201156.s002.tif]

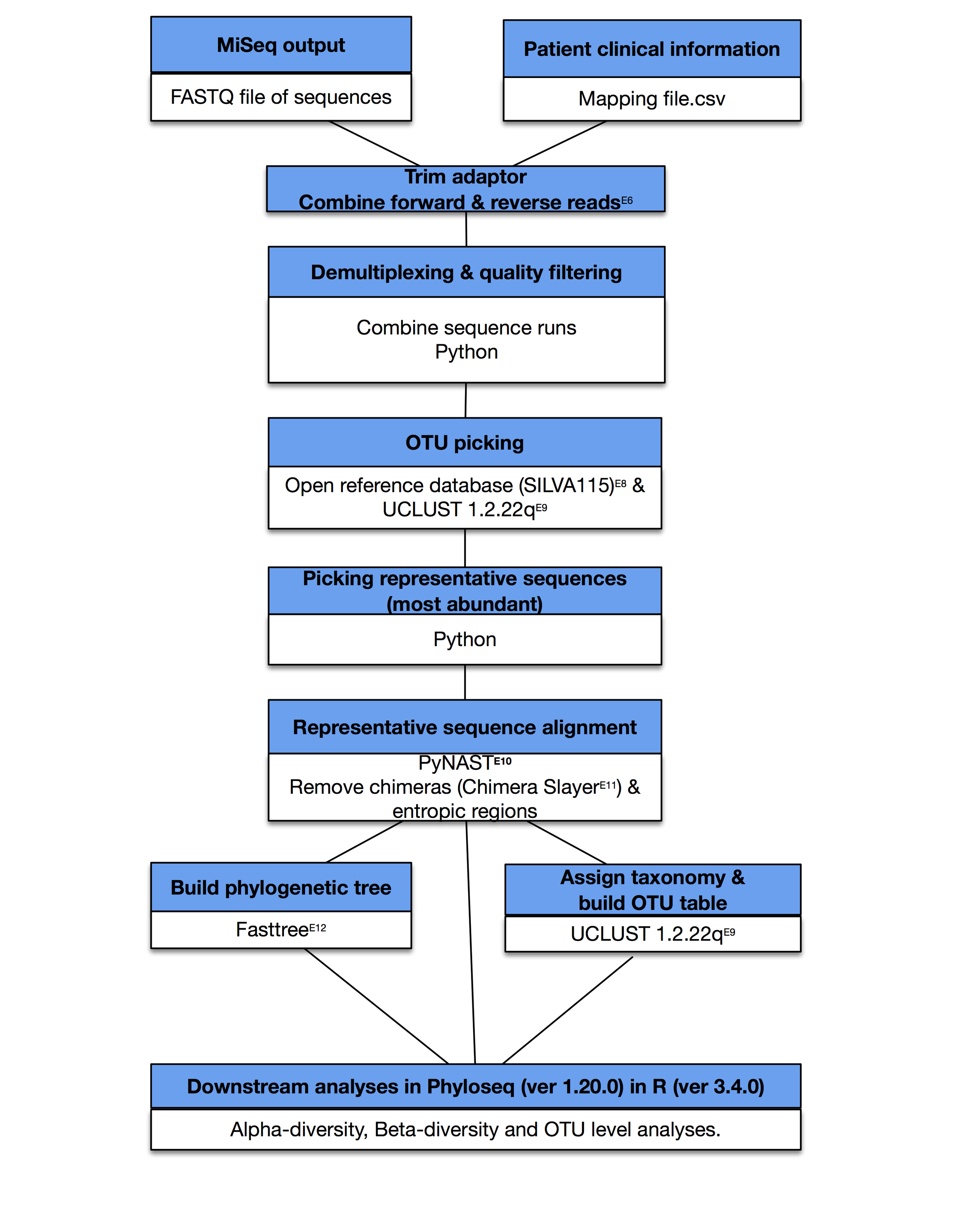

Supplement: S2 Fig — Upstream analyses were performed in QIIME and downstream analyses were performed in Phyloseq in R. (TIF) [file pone.0201156.s003.tif]

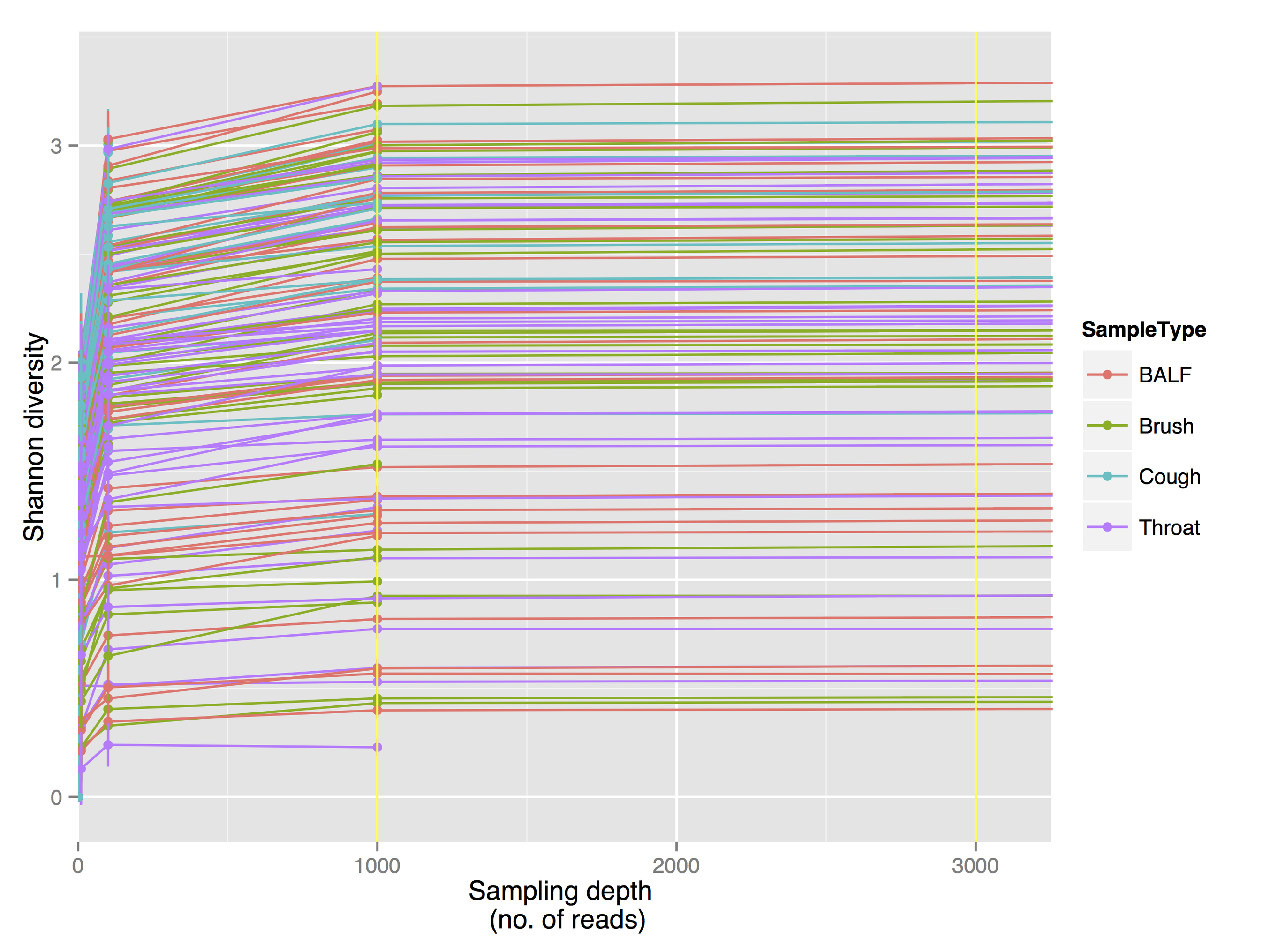

Supplement: S3 Fig — This illustrates that an asymptote is reached by 1,000 reads. At this threshold, the majority of OTUs have been sampled and little additional information is obtained at higher rarefaction levels. Consequently a rarefaction level of 1,000 reads was chosen. (TIF) [file pone.0201156.s004.tif]

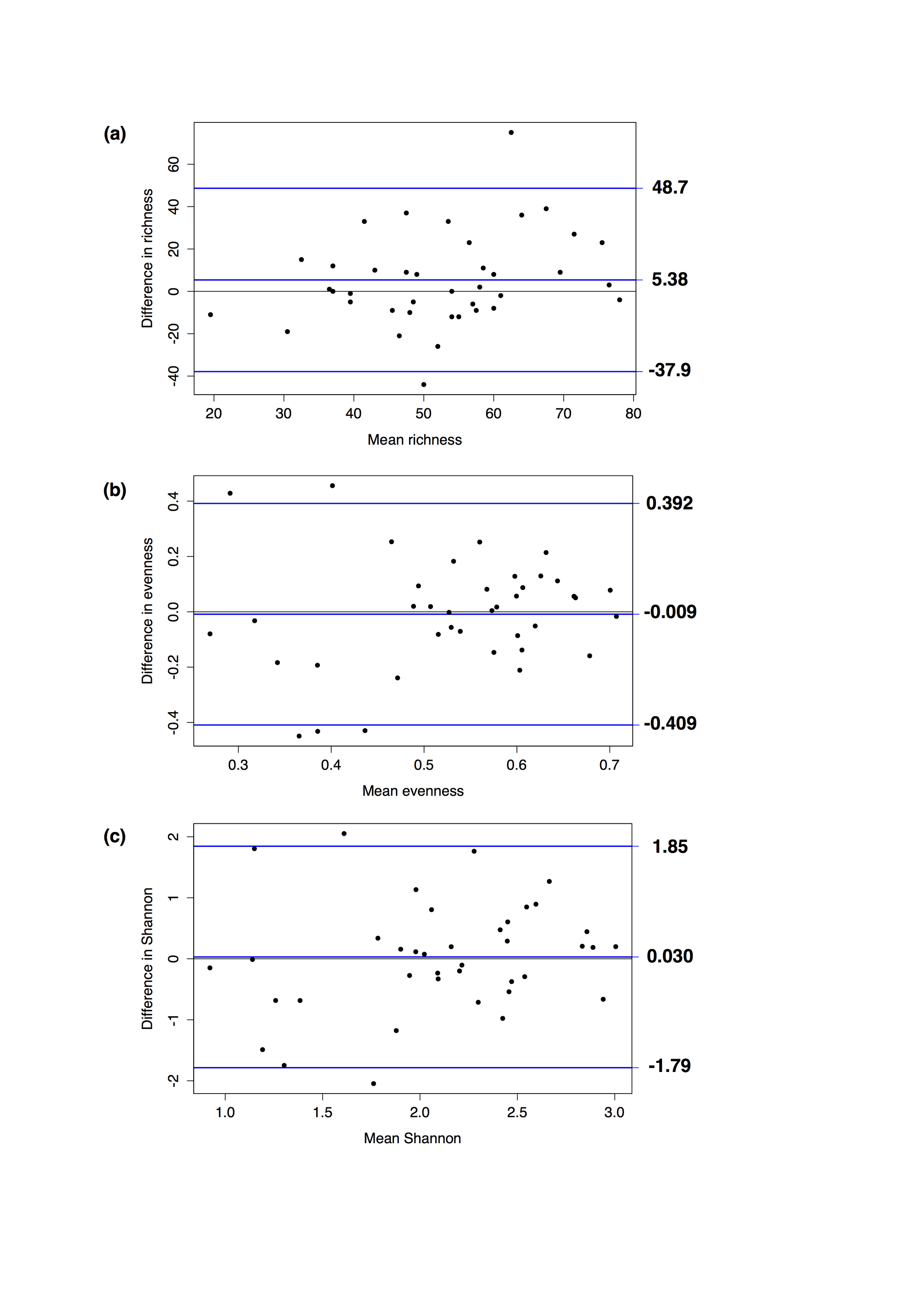

Supplement: S5 Fig — Overall agreement is seen between samples, apart from at low levels of evenness and Shannon Diversity. (TIF) [file pone.0201156.s006.tif]
