# Supplementary material for: Comparison of the upper and lower airway microbiota in children with chronic lung diseases
Source: PLoS One. 2018 Aug 2;13(8):e0201156. doi: 10.1371/journal.pone.0201156 (PMC6071972; doi:10.1371/journal.pone.0201156)
Supplement: S4 Fig — Only BALF samples were sent for bacterial culture as part of routine clinical care. The results of BALF culture and disease group are also detailed. (PDF) [file pone.0201156.s005.pdf]

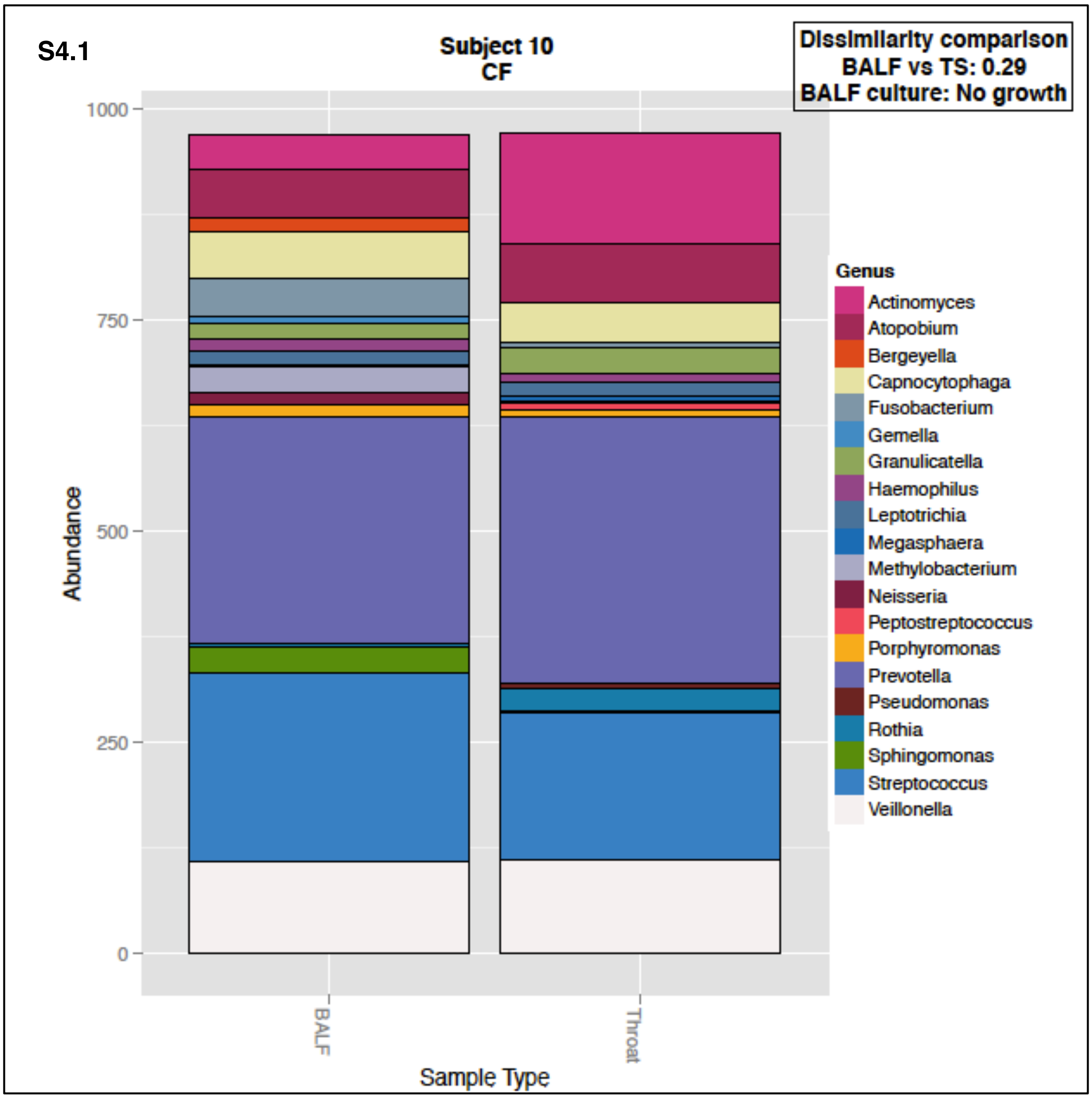

S4.1
Subject 10
CF
Dissimilarity comparison
BALF vs TS: 0.29
BALF culture: No growth
1000
750
500
250
0
Abundance
BALF
Throat
Sample Type
Genus
Actinomyces
Atopobium
Bergeyella
Capnocytophaga
Fusobacterium
Gemella
Granulicatella
Haemophilus
Leptotrichia
Megasphaera
Methylobacterium
Neisseria
Peptostreptococcus
Porphyromonas
Prevotella
Pseudomonas
Rothia
Sphingomonas
Streptococcus
Veillonella

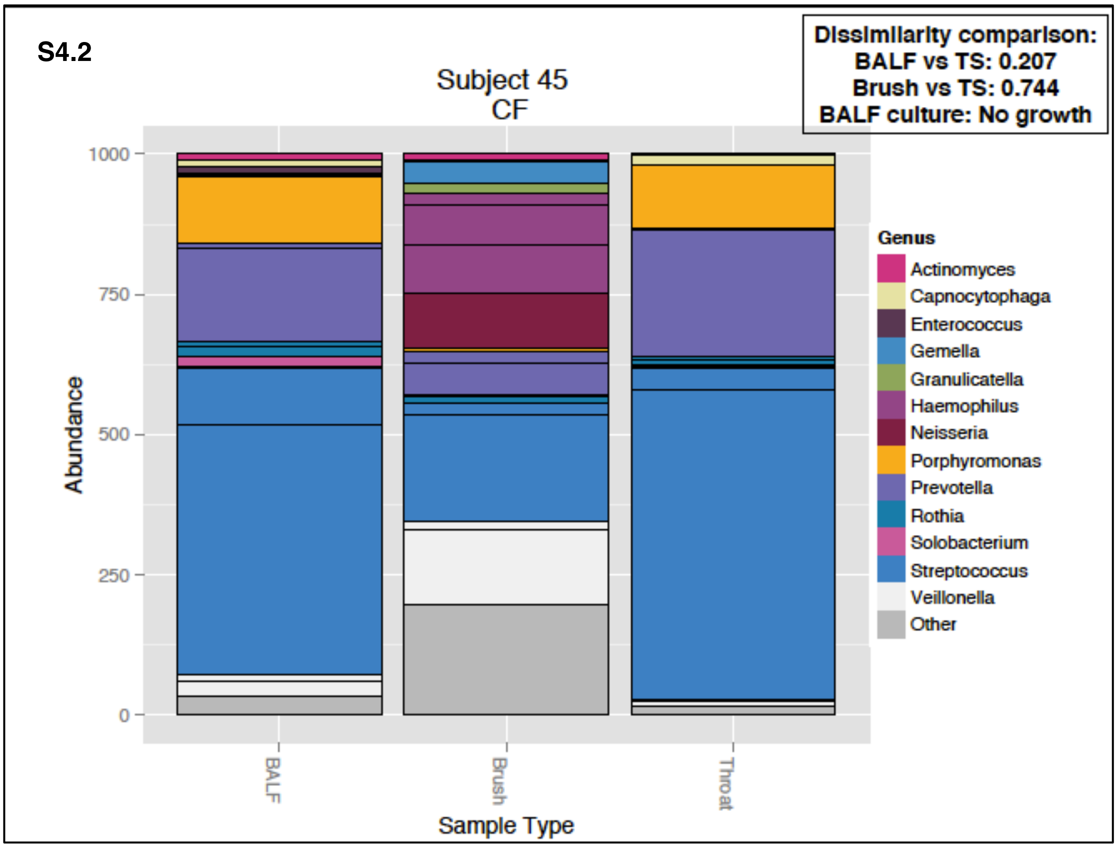
S4.2
Subject 45
CF
Dissimilarity comparison:
BALF vs TS: 0.207
Brush vs TS: 0.744
BALF culture: No growth
Abundance
1000
750
500
250
0
BALF
Brush
Throat
Sample Type
Genus
Actinomyces
Capnocytophaga
Enterococcus
Gemella
Granulicatella
Haemophilus
Neisseria
Porphyromonas
Prevotella
Rothia
Solobacterium
Streptococcus
Veillonella
Other

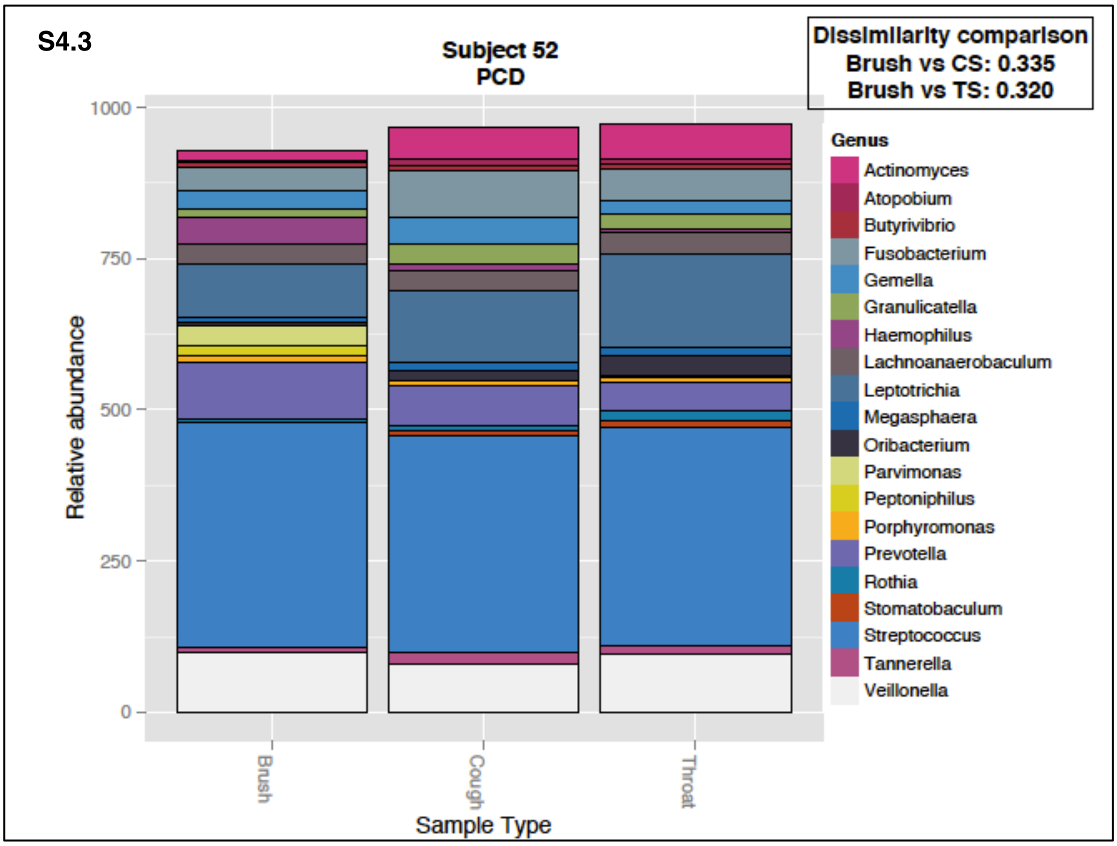
S4.3
Subject 52
PCD
Dissimilarity comparison
Brush vs CS: 0.335
Brush vs TS: 0.320
Relative abundance
1000
750
500
250
0
Brush
Cough
Throat
Sample Type
Genus
Actinomyces
Atopobium
Butyrivibrio
Fusobacterium
Gemella
Granulicatella
Haemophilus
Lachnoanaerobaculum
Leptotrichia
Megasphaera
Oribacterium
Parvimonas
Peptoniphilus
Porphyromonas
Prevotella
Rothia
Stomatobaculum
Streptococcus
Tannerella
Veillonella

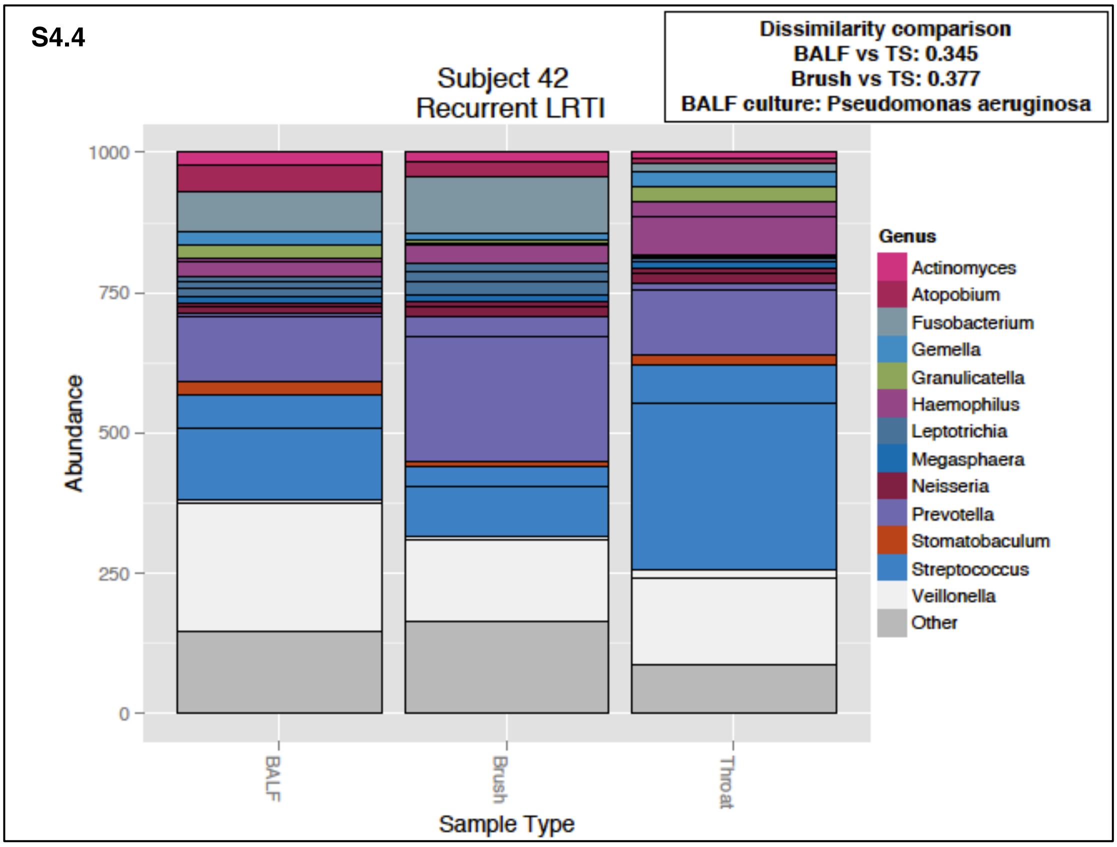
S4.4
Subject 42
Recurrent LRTI
Dissimilarity comparison
BALF vs TS: 0.345
Brush vs TS: 0.377
BALF culture: Pseudomonas aeruginosa
Abundance
1000
750
500
250
0
BALF
Brush
Throat
Sample Type
Genus
Actinomyces
Atopobium
Fusobacterium
Gemella
Granulicatella
Haemophilus
Leptotrichia
Megasphaera
Neisseria
Prevotella
Stomatobaculum
Streptococcus
Veillonella
Other

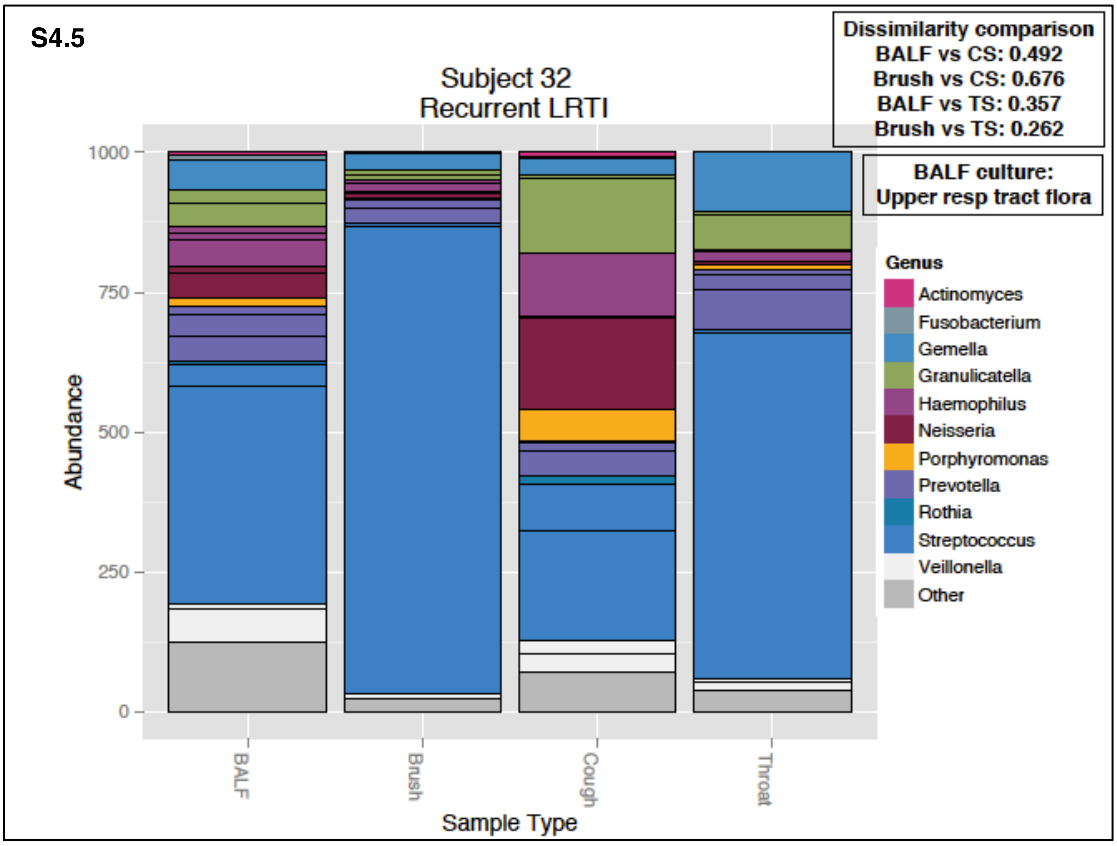
S4.5
Subject 32
Recurrent LRTI
Dissimilarity comparison
BALF vs CS: 0.492
Brush vs CS: 0.676
BALF vs TS: 0.357
Brush vs TS: 0.262
BALF culture:
Upper resp tract flora
Abundance
1000
750
500
250
0
BALF
Brush
Cough
Throat
Sample Type
Genus
Actinomyces
Fusobacterium
Gemella
Granulicatella
Haemophilus
Neisseria
Porphyromonas
Prevotella
Rothia
Streptococcus
Veillonella
Other

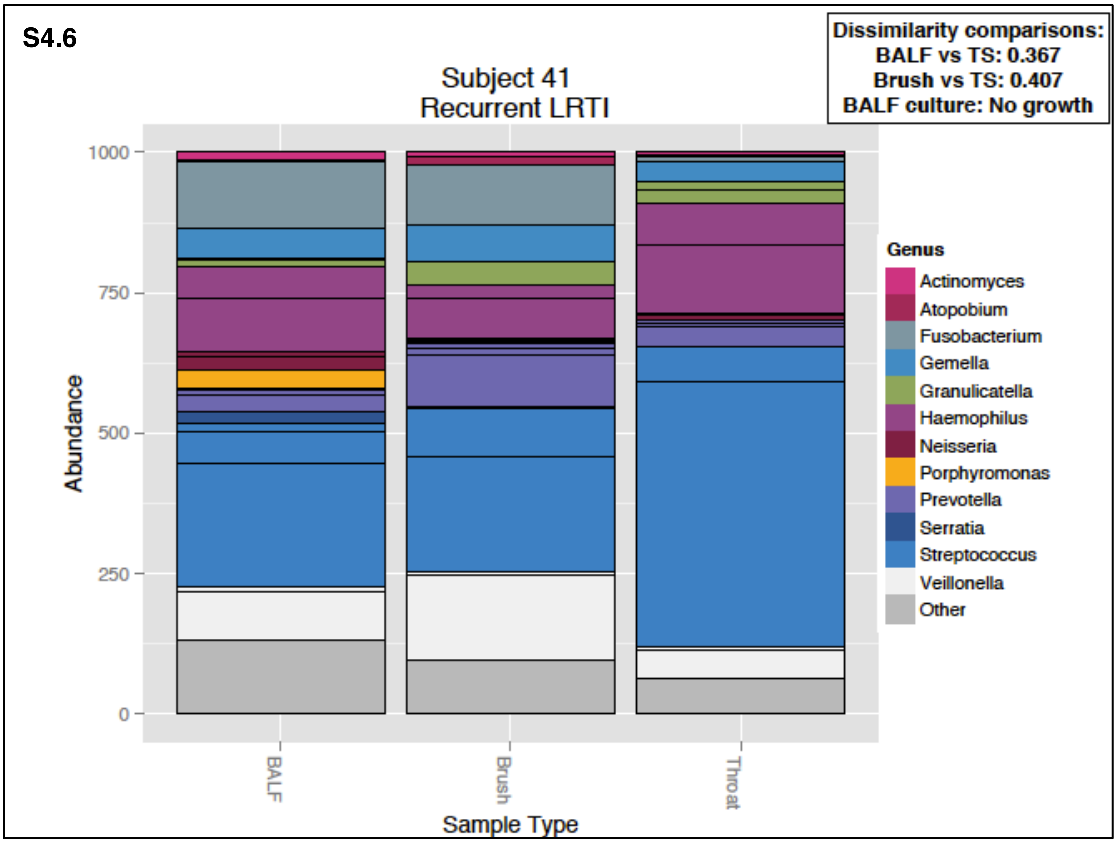
S4.6
Dissimilarity comparisons:
BALF vs TS: 0.367
Brush vs TS: 0.407
BALF culture: No growth
Subject 41
Recurrent LRTI
Abundance
1000
750
500
250
0
Genus
Actinomyces
Atopobium
Fusobacterium
Gemella
Granulicatella
Haemophilus
Neisseria
Porphyromonas
Prevotella
Serratia
Streptococcus
Veillonella
Other
BALF
Brush
Throat
Sample Type

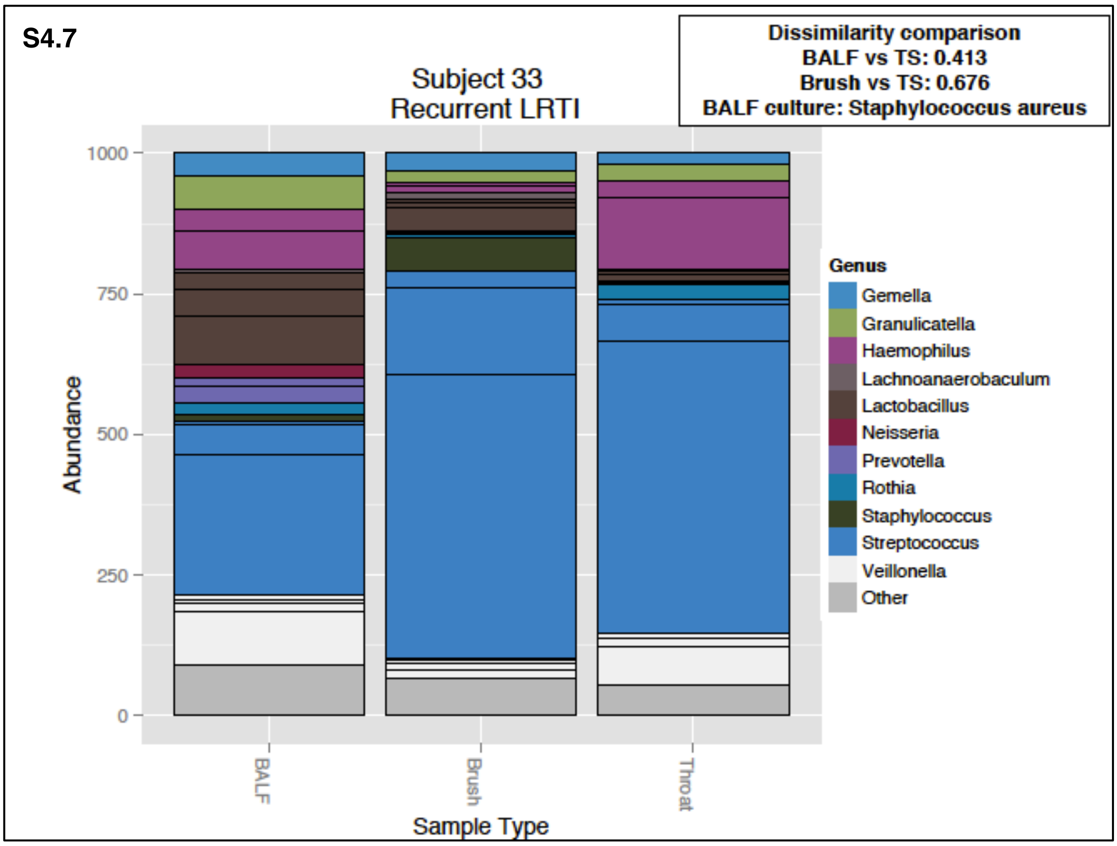
S4.7
Dissimilarity comparison
BALF vs TS: 0.413
Brush vs TS: 0.676
BALF culture: Staphylococcus aureus
Subject 33
Recurrent LRTI
Abundance
1000
750
500
250
0
Genus
Gemella
Granulicatella
Haemophilus
Lachnoanaerobaculum
Lactobacillus
Neisseria
Prevotella
Rothia
Staphylococcus
Streptococcus
Veillonella
Other
BALF
Brush
Throat
Sample Type

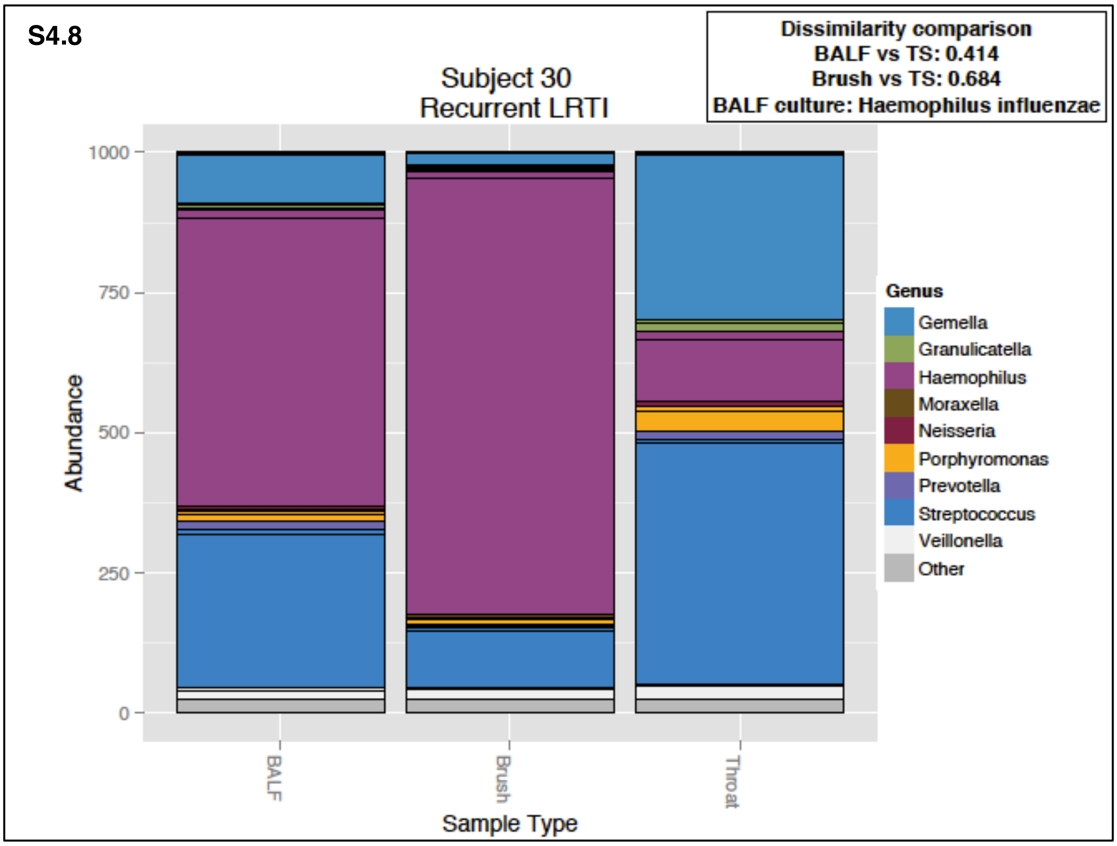
S4.8
Subject 30
Recurrent LRTI
Dissimilarity comparison
BALF vs TS: 0.414
Brush vs TS: 0.684
BALF culture: Haemophilus influenzae
1000
750
500
250
0
Abundance
BALF
Brush
Throat
Sample Type
Genus
Gemella
Granulicatella
Haemophilus
Moraxella
Neisseria
Porphyromonas
Prevotella
Streptococcus
Veillonella
Other

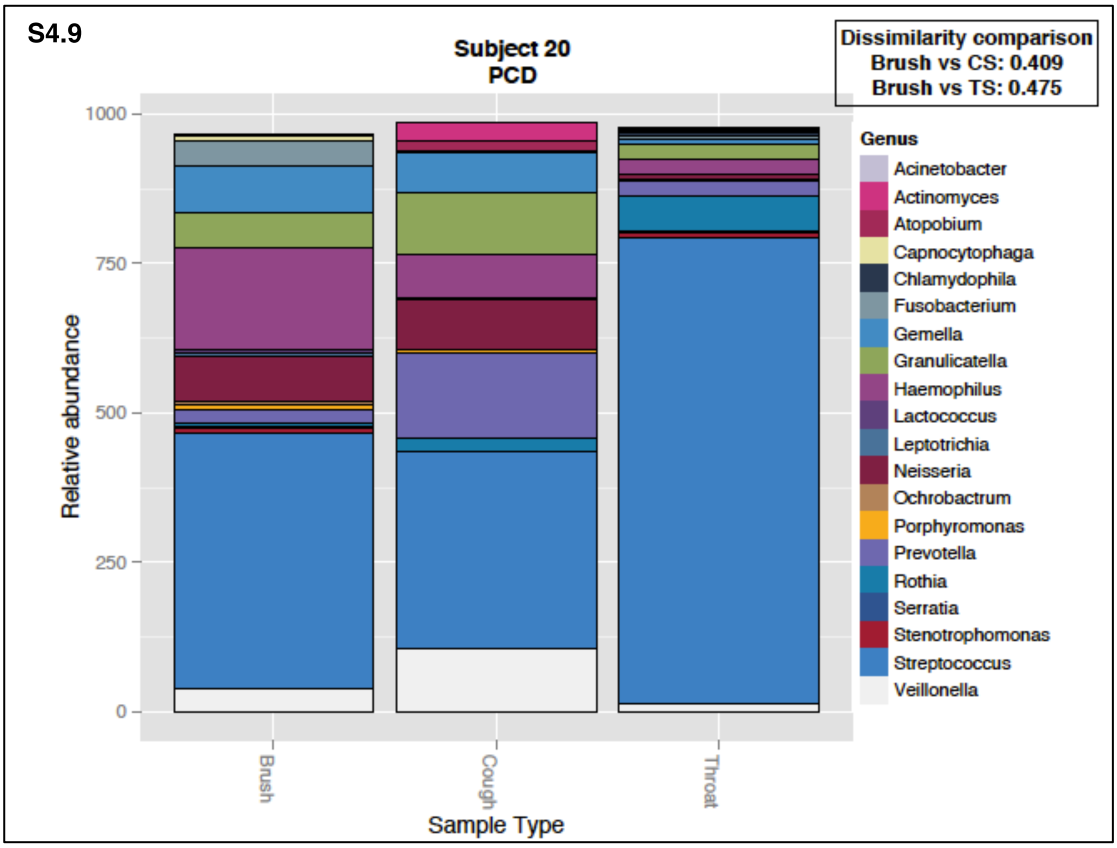
S4.9
Subject 20
PCD
Dissimilarity comparison
Brush vs CS: 0.409
Brush vs TS: 0.475
1000
750
500
250
0
Relative abundance
Brush
Cough
Throat
Sample Type
Genus
Acinetobacter
Actinomyces
Atopobium
Capnocytophaga
Chlamydophila
Fusobacterium
Gemella
Granulicatella
Haemophilus
Lactococcus
Leptotrichia
Neisseria
Ochrobactrum
Porphyromonas
Prevotella
Rothia
Serratia
Stenotrophomonas
Streptococcus
Veillonella

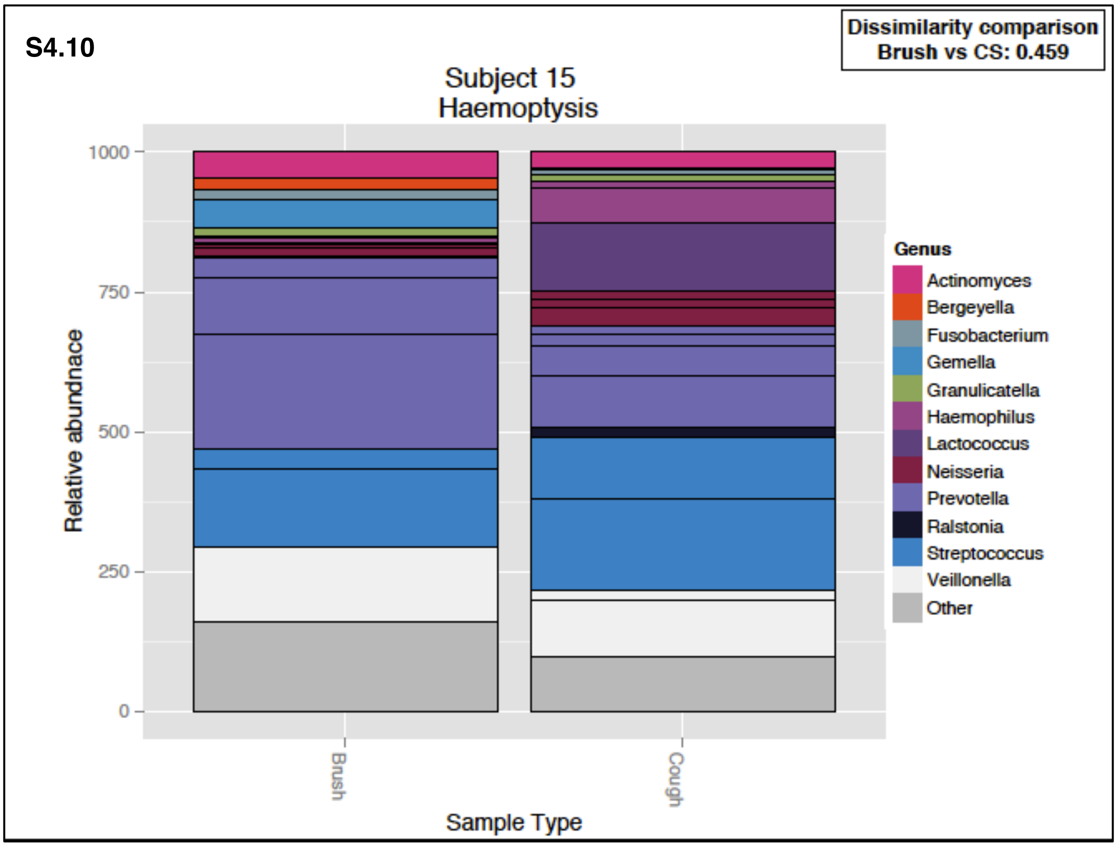
S4.10
Dissimilarity comparison
Brush vs CS: 0.459
Subject 15
Haemoptysis
Relative abundnace
1000
750
500
250
0
Brush
Cough
Sample Type
Genus
Actinomyces
Bergeyella
Fusobacterium
Gemella
Granulicatella
Haemophilus
Lactococcus
Neisseria
Prevotella
Ralstonia
Streptococcus
Veillonella
Other

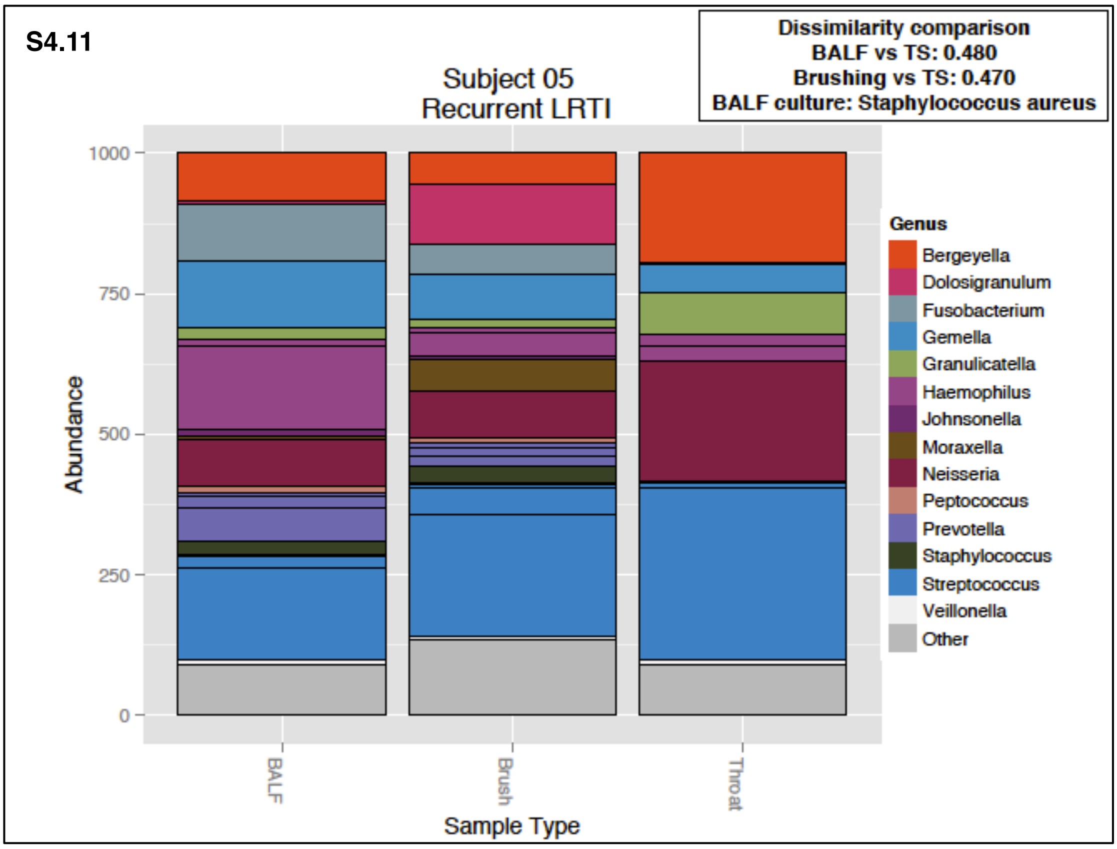
S4.11
Dissimilarity comparison
BALF vs TS: 0.480
Brushing vs TS: 0.470
BALF culture: Staphylococcus aureus
Subject 05
Recurrent LRTI
Abundance
1000
750
500
250
0
BALF
Brush
Throat
Sample Type
Genus
Bergeyella
Dolosigranulum
Fusobacterium
Gemella
Granulicatella
Haemophilus
Johnsonella
Moraxella
Neisseria
Peptococcus
Prevotella
Staphylococcus
Streptococcus
Veillonella
Other

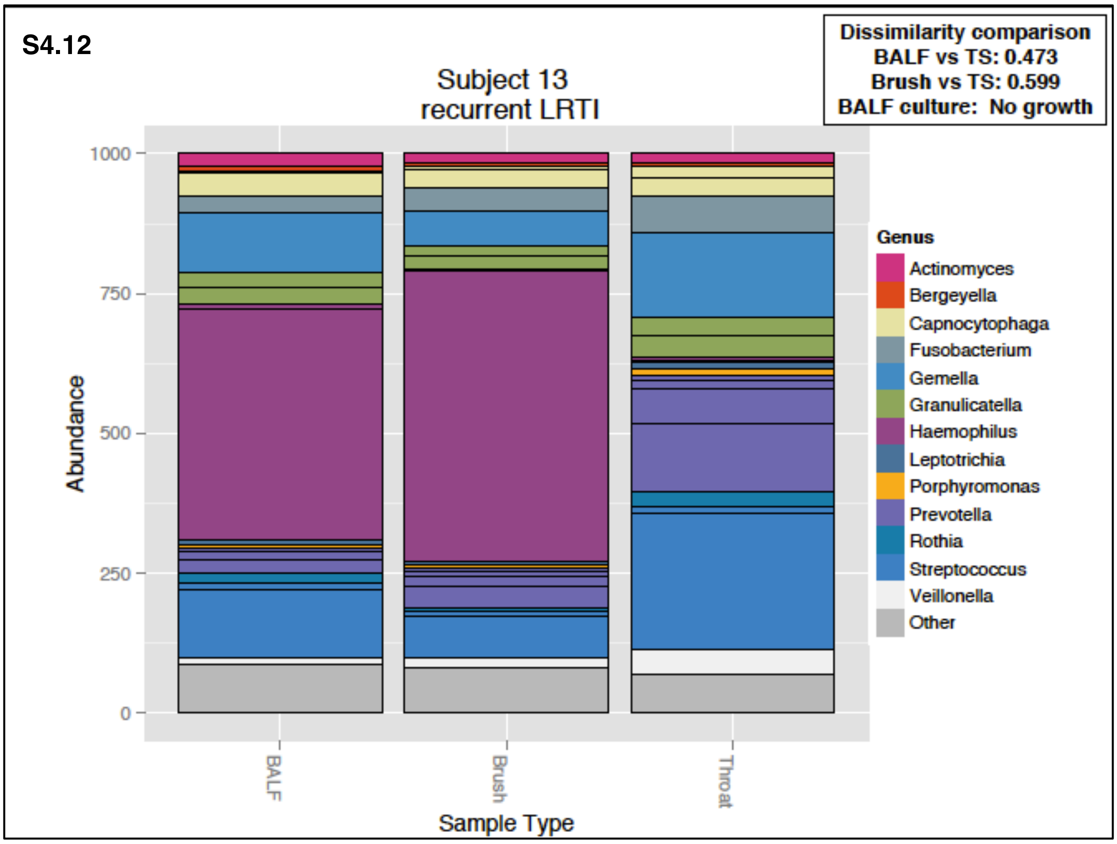
S4.12
Subject 13
recurrent LRTI
Dissimilarity comparison
BALF vs TS: 0.473
Brush vs TS: 0.599
BALF culture: No growth
Abundance
1000
750
500
250
0
BALF
Brush
Throat
Sample Type
Genus
Actinomyces
Bergeyella
Capnocytophaga
Fusobacterium
Gemella
Granulicatella
Haemophilus
Leptotrichia
Porphyromonas
Prevotella
Rothia
Streptococcus
Veillonella
Other

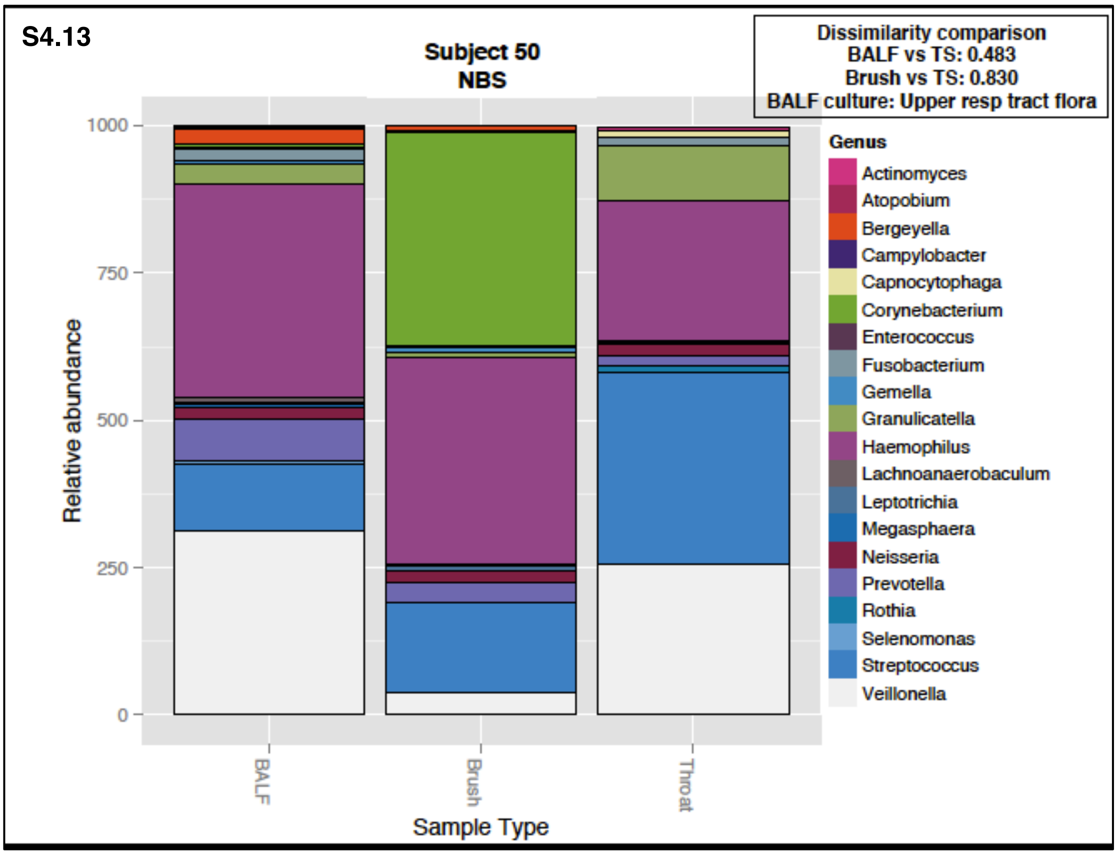
S4.13
Subject 50
NBS
Dissimilarity comparison
BALF vs TS: 0.483
Brush vs TS: 0.830
BALF culture: Upper resp tract flora
Relative abundance
1000
750
500
250
0
BALF
Brush
Throat
Sample Type
Genus
Actinomyces
Atopobium
Bergeyella
Campylobacter
Capnocytophaga
Corynebacterium
Enterococcus
Fusobacterium
Gemella
Granulicatella
Haemophilus
Lachnoanaerobaculum
Leptotrichia
Megasphaera
Neisseria
Prevotella
Rothia
Selenomonas
Streptococcus
Veillonella

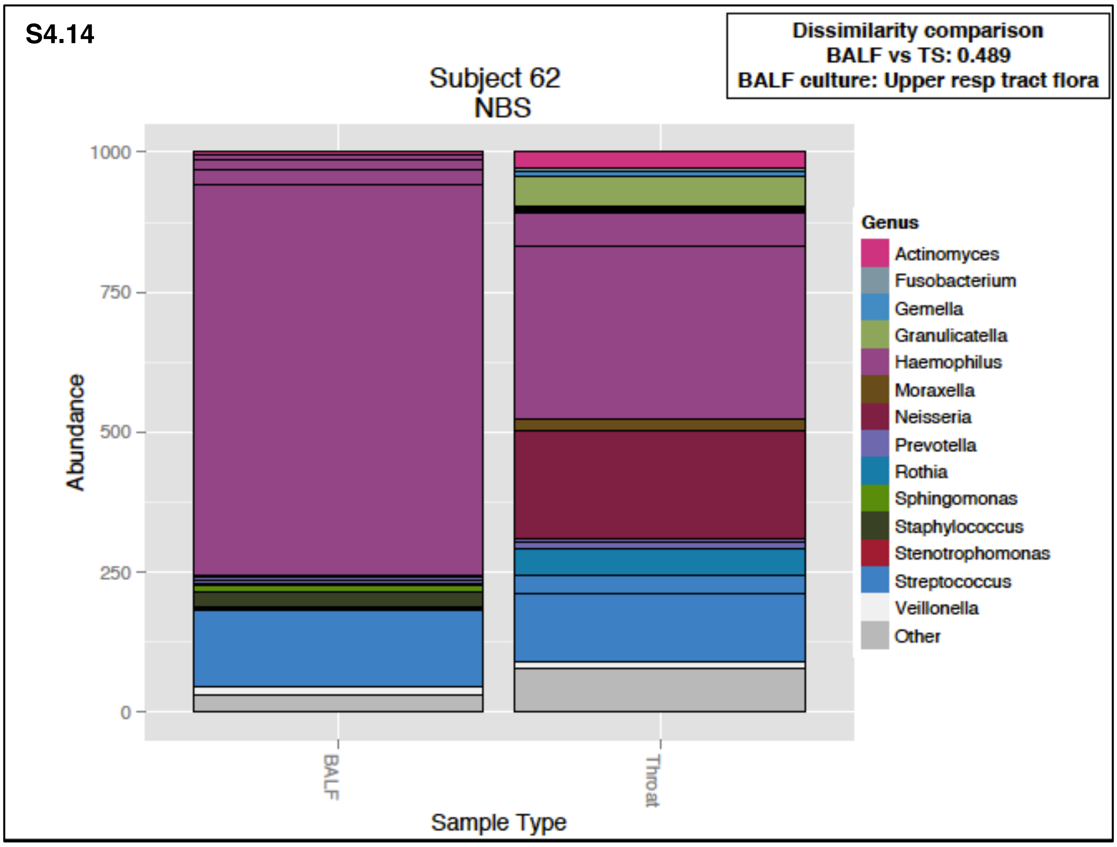
S4.14
Dissimilarity comparison
BALF vs TS: 0.489
BALF culture: Upper resp tract flora
Subject 62
NBS
Abundance
1000
750
500
250
0
BALF
Throat
Sample Type
Genus
Actinomyces
Fusobacterium
Gemella
Granulicatella
Haemophilus
Moraxella
Neisseria
Prevotella
Rothia
Sphingomonas
Staphylococcus
Stenotrophomonas
Streptococcus
Veillonella
Other

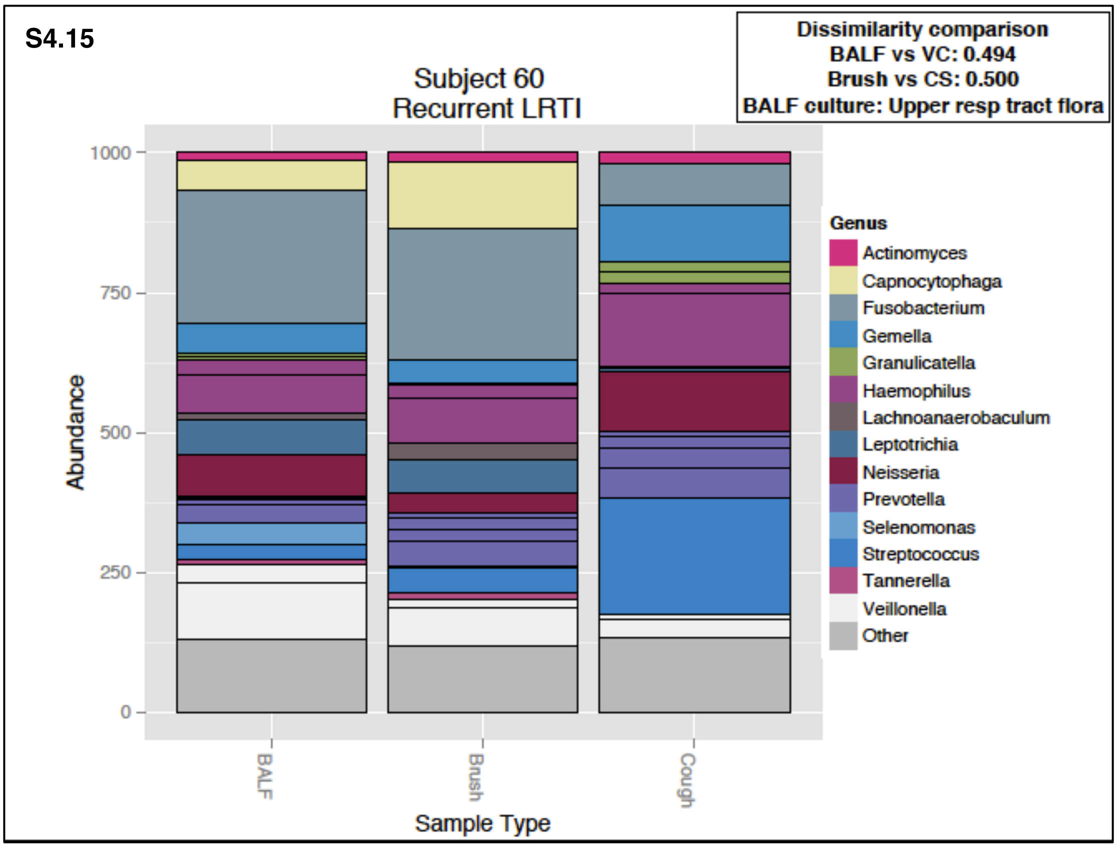
S4.15
Dissimilarity comparison
BALF vs VC: 0.494
Brush vs CS: 0.500
BALF culture: Upper resp tract flora
Subject 60
Recurrent LRTI
Abundance
1000
750
500
250
0
BALF
Brush
Cough
Sample Type
Genus
Actinomyces
Capnocytophaga
Fusobacterium
Gemella
Granulicatella
Haemophilus
Lachnoanaerobaculum
Leptotrichia
Neisseria
Prevotella
Selenomonas
Streptococcus
Tannerella
Veillonella
Other

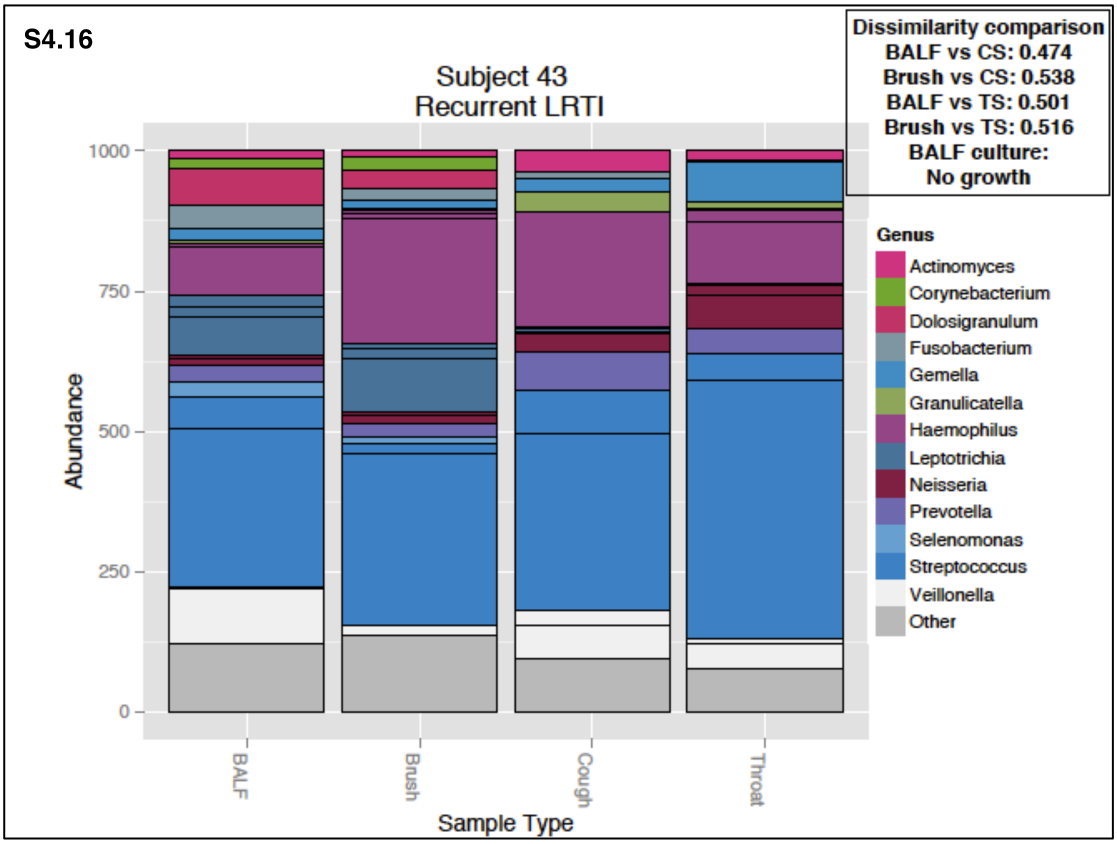
S4.16
Subject 43
Recurrent LRTI
Dissimilarity comparison
BALF vs CS: 0.474
Brush vs CS: 0.538
BALF vs TS: 0.501
Brush vs TS: 0.516
BALF culture:
No growth
Abundance
1000
750
500
250
0
BALF
Brush
Cough
Throat
Sample Type
Genus
Actinomyces
Corynebacterium
Dolosigranulum
Fusobacterium
Gemella
Granulicatella
Haemophilus
Leptotrichia
Neisseria
Prevotella
Selenomonas
Streptococcus
Veillonella
Other

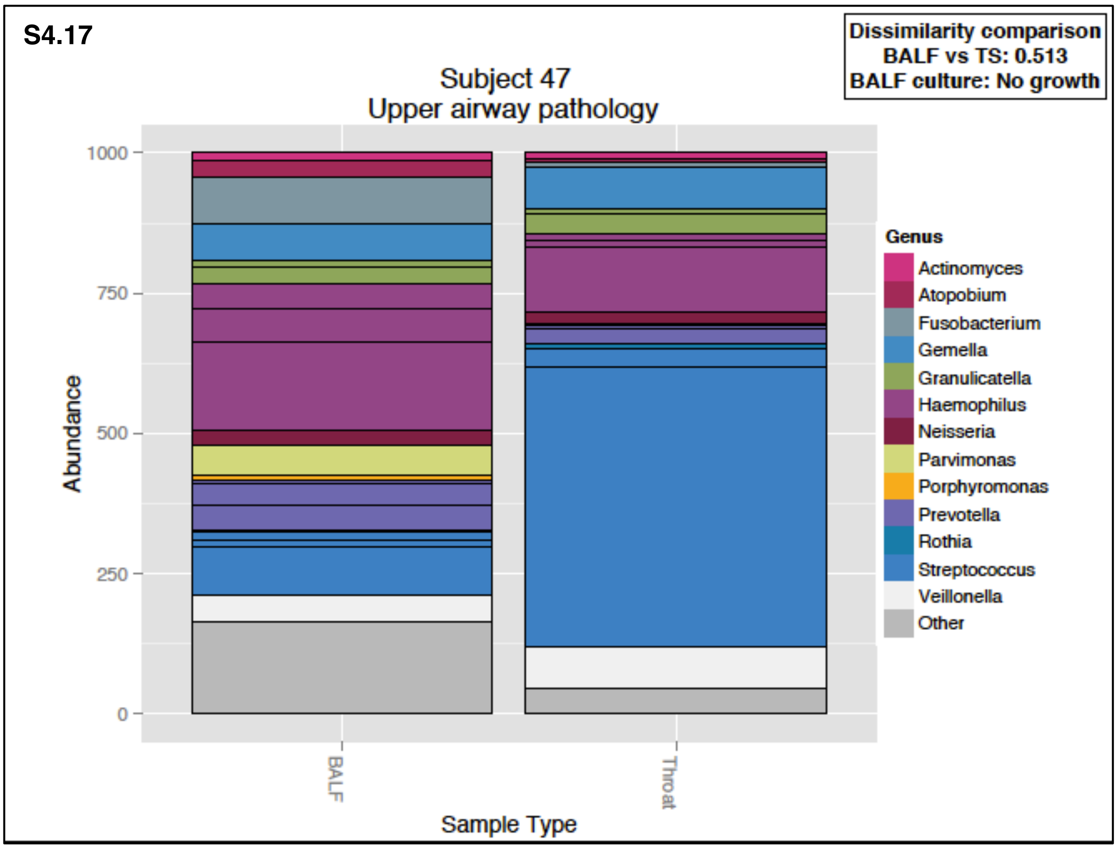
S4.17
Subject 47
Upper airway pathology
Dissimilarity comparison
BALF vs TS: 0.513
BALF culture: No growth
Abundance
1000
750
500
250
0
BALF
Throat
Sample Type
Genus
Actinomyces
Atopobium
Fusobacterium
Gemella
Granulicatella
Haemophilus
Neisseria
Parvimonas
Porphyromonas
Prevotella
Rothia
Streptococcus
Veillonella
Other

S4.18

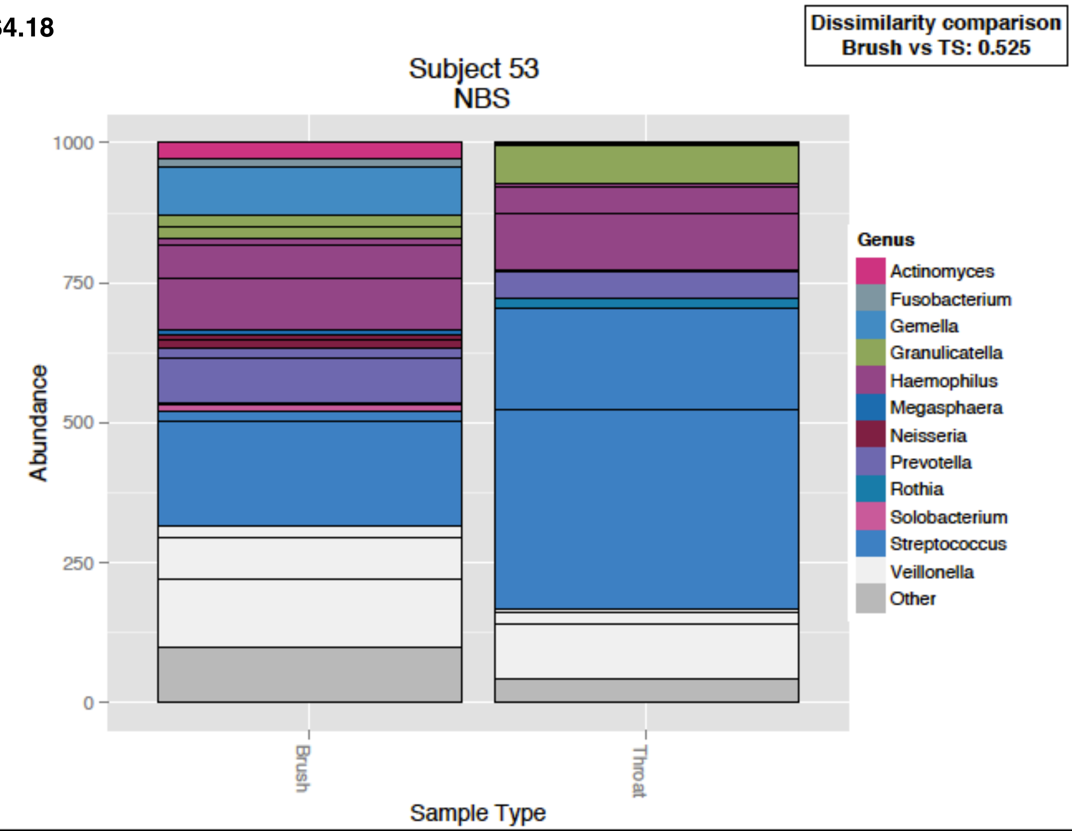
Dissimilarity comparison
Brush vs TS: 0.525
Subject 53
NBS
1000
750
500
250
0
Abundance
Brush
Throat
Sample Type
Genus
Actinomyces
Fusobacterium
Gemella
Granulicatella
Haemophilus
Megasphaera
Neisseria
Prevotella
Rothia
Solobacterium
Streptococcus
Veillonella
Other

S4.19

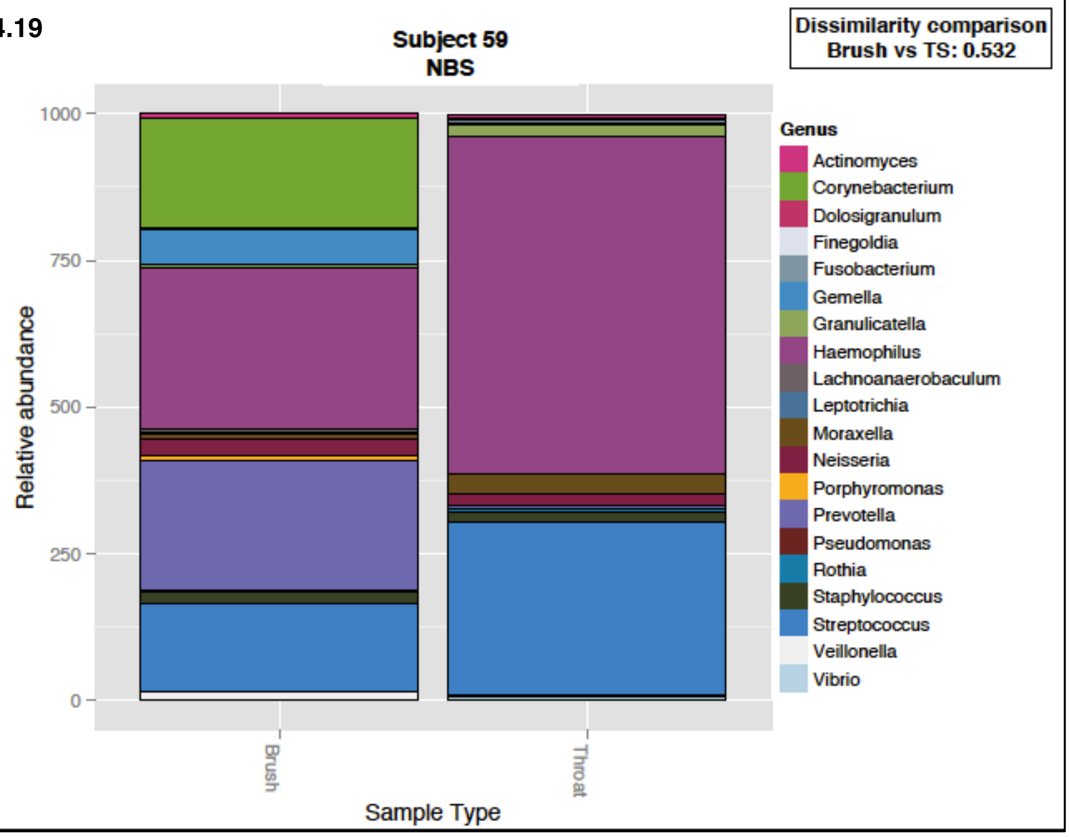
Dissimilarity comparison
Brush vs TS: 0.532
Subject 59
NBS
1000
750
500
250
0
Relative abundance
Brush
Throat
Sample Type
Genus
Actinomyces
Corynebacterium
Dolosigranulum
Finegoldia
Fusobacterium
Gemella
Granulicatella
Haemophilus
Lachnoanaerobaculum
Leptotrichia
Moraxella
Neisseria
Porphyromonas
Prevotella
Pseudomonas
Rothia
Staphylococcus
Streptococcus
Veillonella
Vibrio

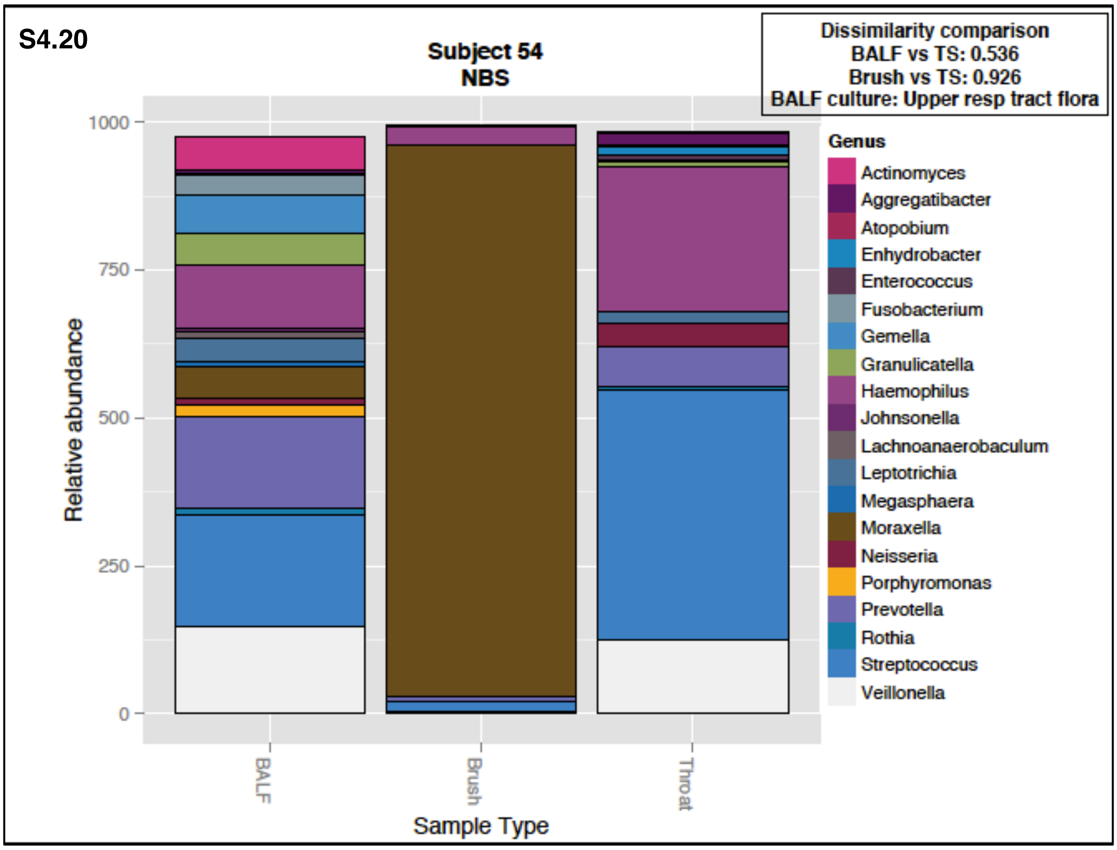

S4.20
Subject 54
NBS
Dissimilarity comparison
BALF vs TS: 0.536
Brush vs TS: 0.926
BALF culture: Upper resp tract flora
Relative abundance
1000
750
500
250
0
BALF
Brush
Throat
Sample Type
Genus
Actinomyces
Aggregatibacter
Atopobium
Enhydrobacter
Enterococcus
Fusobacterium
Gemella
Granulicatella
Haemophilus
Johnsonella
Lachnoanaerobaculum
Leptotrichia
Megasphaera
Moraxella
Neisseria
Porphyromonas
Prevotella
Rothia
Streptococcus
Veillonella


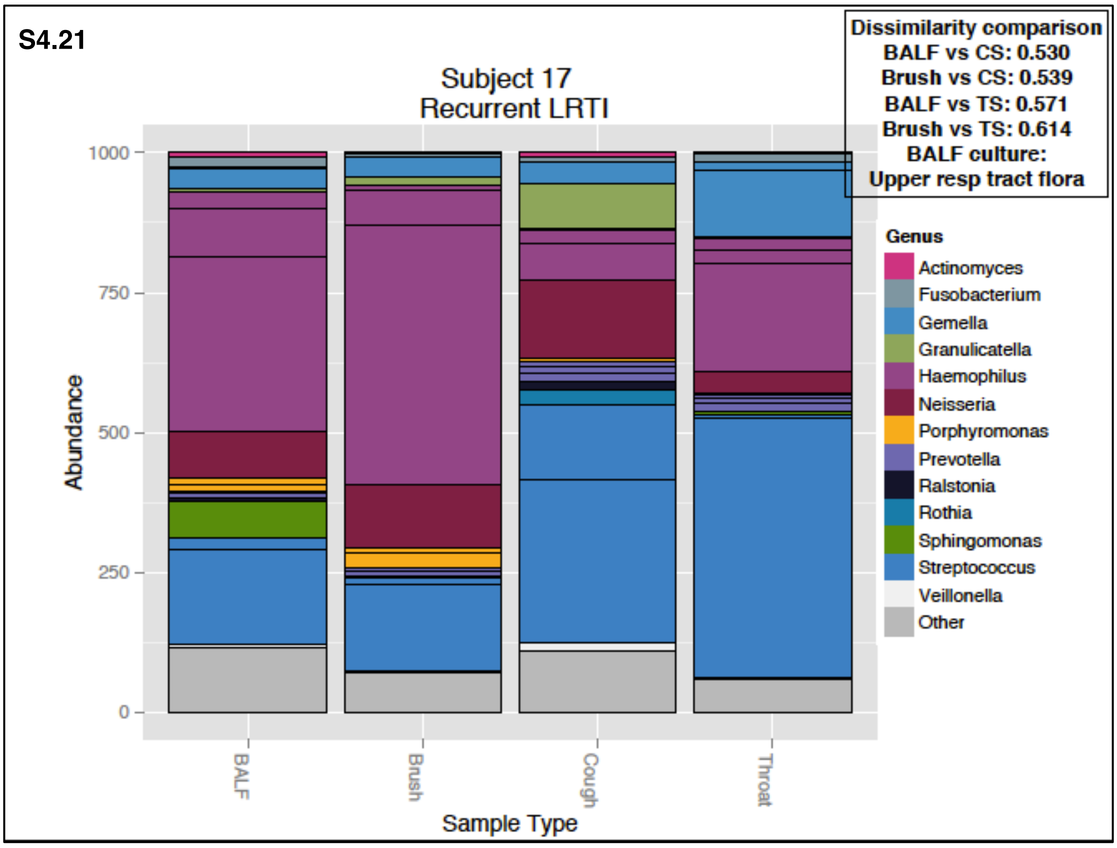

S4.21
Subject 17
Recurrent LRTI
Dissimilarity comparison
BALF vs CS: 0.530
Brush vs CS: 0.539
BALF vs TS: 0.571
Brush vs TS: 0.614
BALF culture:
Upper resp tract flora
Abundance
1000
750
500
250
0
BALF
Brush
Cough
Throat
Sample Type
Genus
Actinomyces
Fusobacterium
Gemella
Granulicatella
Haemophilus
Neisseria
Porphyromonas
Prevotella
Ralstonia
Rothia
Sphingomonas
Streptococcus
Veillonella
Other

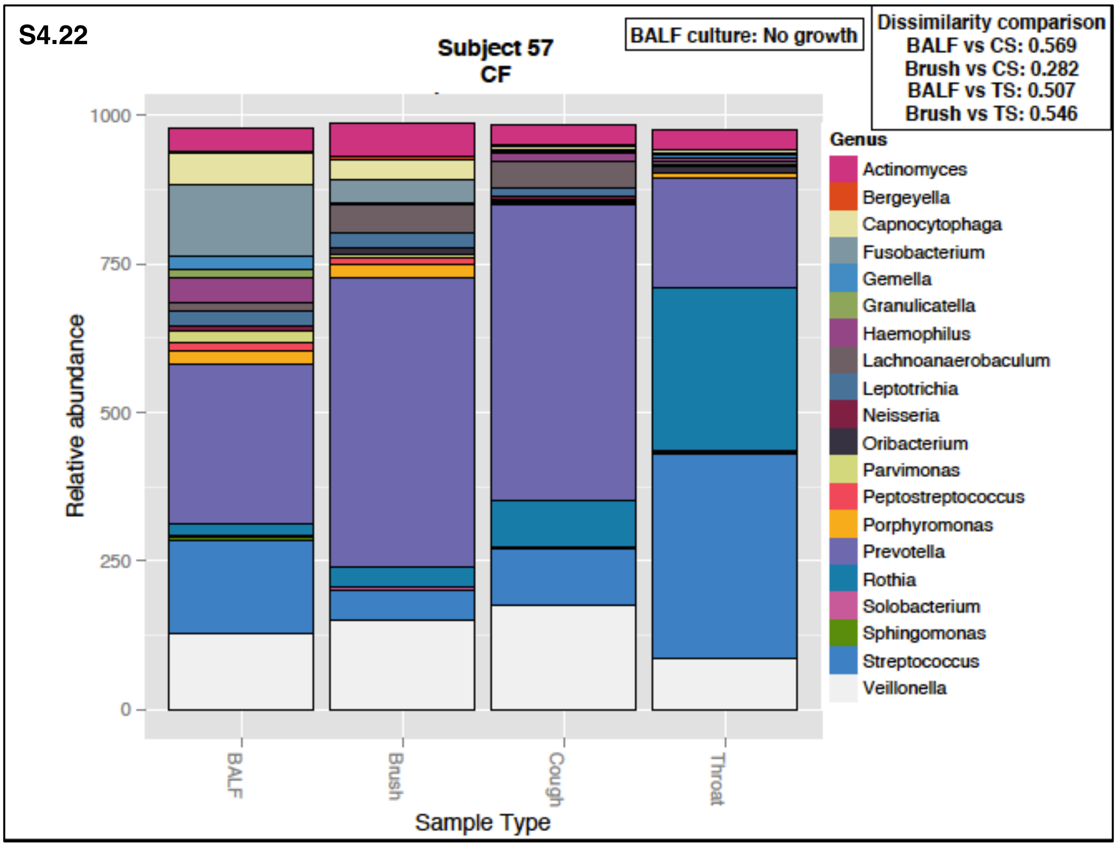
S4.22
Subject 57
CF
BALF culture: No growth
Dissimilarity comparison
BALF vs CS: 0.569
Brush vs CS: 0.282
BALF vs TS: 0.507
Brush vs TS: 0.546
Relative abundance
1000
750
500
250
0
BALF
Brush
Cough
Throat
Sample Type
Genus
Actinomyces
Bergeyella
Capnocytophaga
Fusobacterium
Gemella
Granulicatella
Haemophilus
Lachnoanaerobaculum
Leptotrichia
Neisseria
Oribacterium
Parvimonas
Peptostreptococcus
Porphyromonas
Prevotella
Rothia
Solobacterium
Sphingomonas
Streptococcus
Veillonella

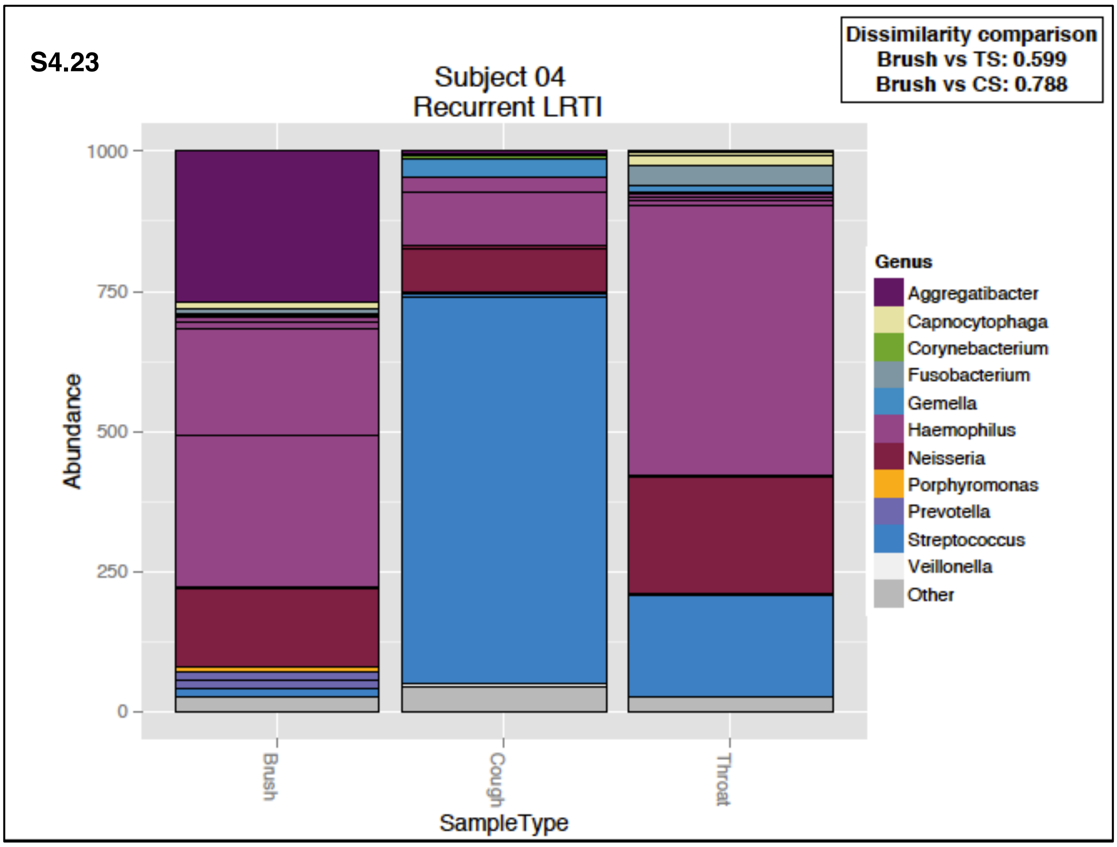
S4.23
Subject 04
Recurrent LRTI
Dissimilarity comparison
Brush vs TS: 0.599
Brush vs CS: 0.788
Abundance
1000
750
500
250
0
Brush
Cough
Throat
SampleType
Genus
Aggregatibacter
Capnocytophaga
Corynebacterium
Fusobacterium
Gemella
Haemophilus
Neisseria
Porphyromonas
Prevotella
Streptococcus
Veillonella
Other

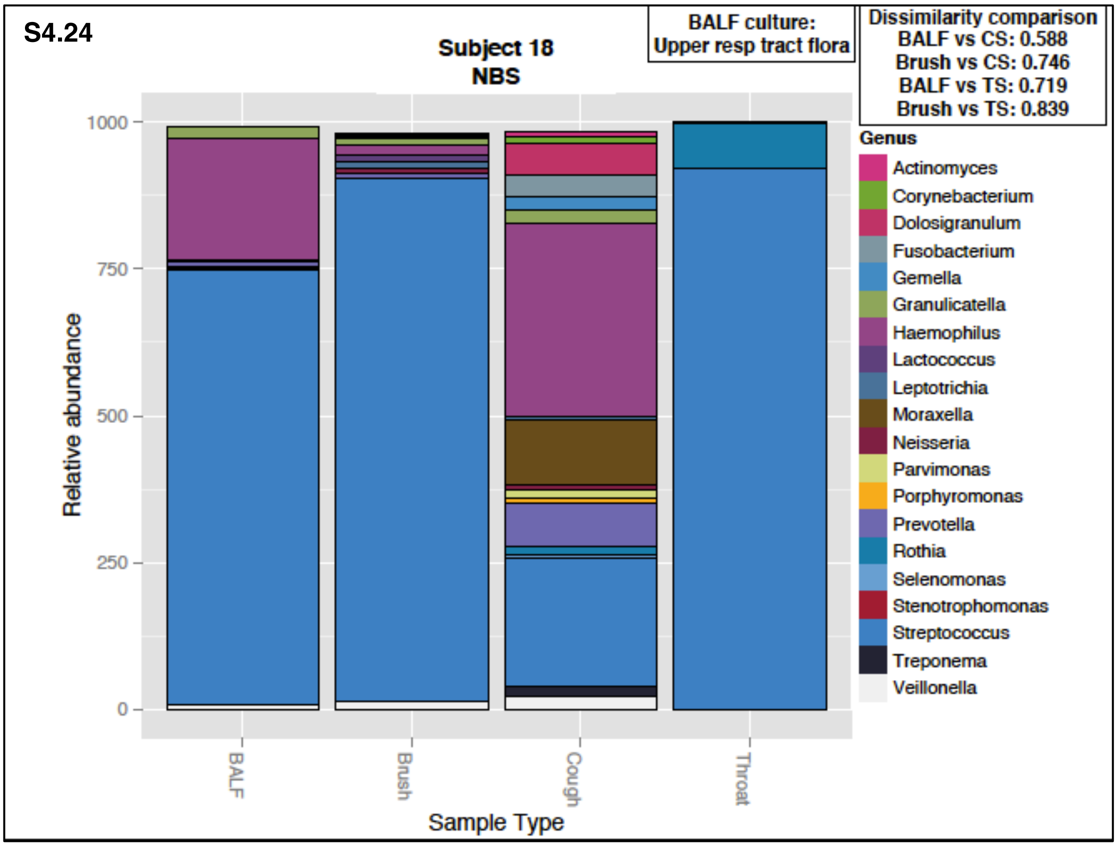

S4.24
Subject 18
NBS
BALF culture:
Upper resp tract flora
Dissimilarity comparison
BALF vs CS: 0.588
Brush vs CS: 0.746
BALF vs TS: 0.719
Brush vs TS: 0.839
Relative abundance
1000
750
500
250
0
BALF
Brush
Cough
Throat
Sample Type
Genus
Actinomyces
Corynebacterium
Dolosigranulum
Fusobacterium
Gemella
Granulicatella
Haemophilus
Lactococcus
Leptotrichia
Moraxella
Neisseria
Parvimonas
Porphyromonas
Prevotella
Rothia
Selenomonas
Stenotrophomonas
Streptococcus
Treponema
Veillonella


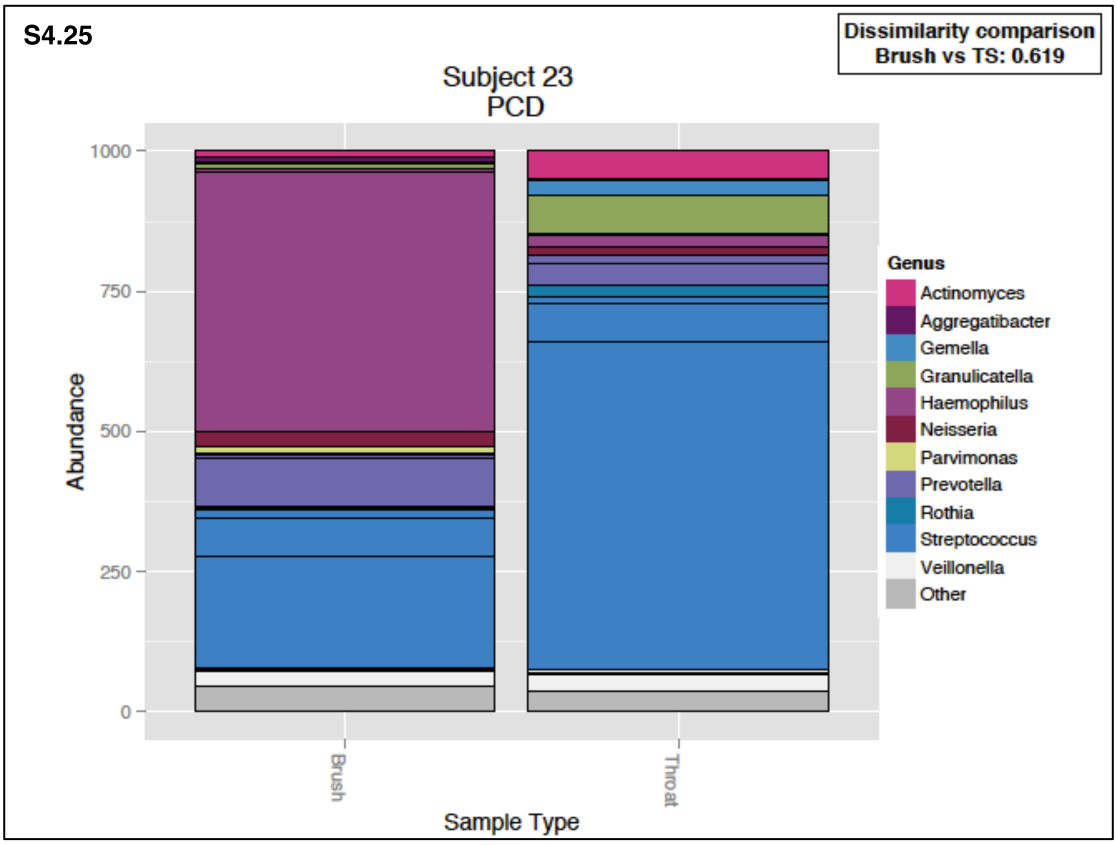

S4.25
Dissimilarity comparison
Brush vs TS: 0.619
Subject 23
PCD
Abundance
1000
750
500
250
0
Brush
Throat
Sample Type
Genus
Actinomyces
Aggregatibacter
Gemella
Granulicatella
Haemophilus
Neisseria
Parvimonas
Prevotella
Rothia
Streptococcus
Veillonella
Other

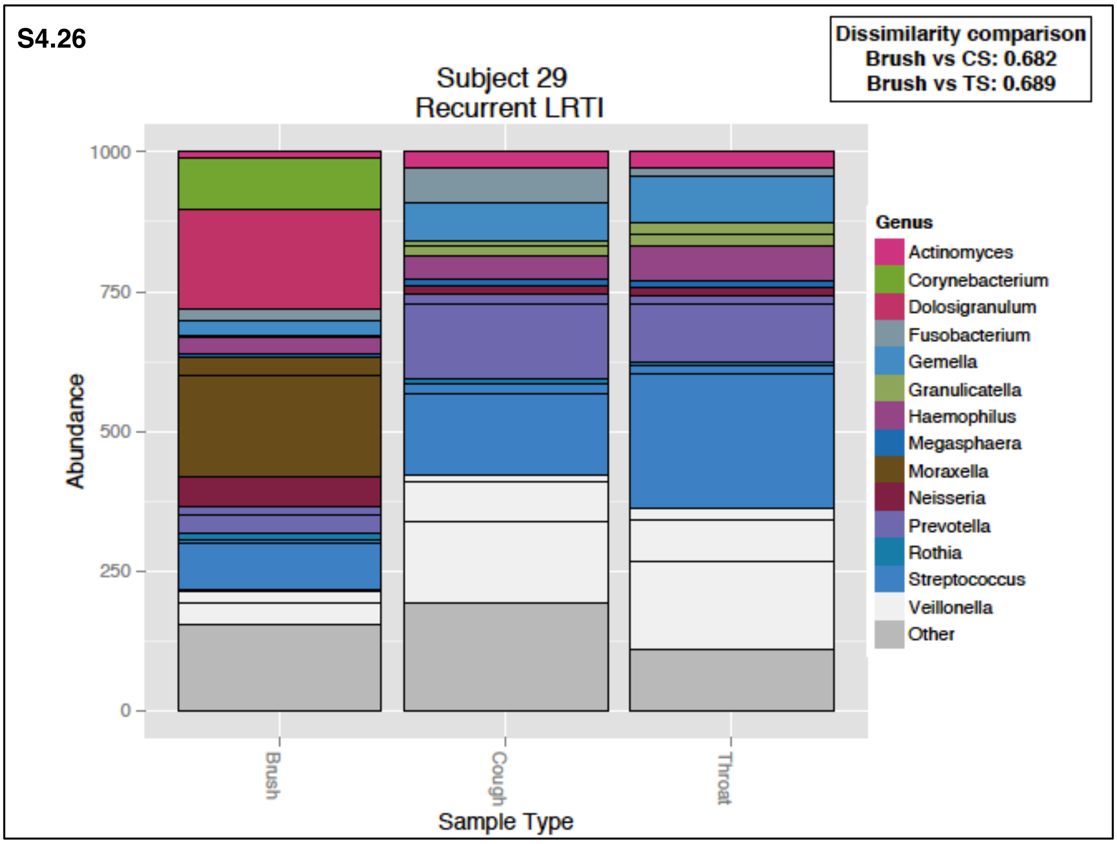

S4.26
Subject 29
Recurrent LRTI
Dissimilarity comparison
Brush vs CS: 0.682
Brush vs TS: 0.689
Abundance
1000
750
500
250
0
Brush
Cough
Throat
Sample Type
Genus
Actinomyces
Corynebacterium
Dolosigranulum
Fusobacterium
Gemella
Granulicatella
Haemophilus
Megasphaera
Moraxella
Neisseria
Prevotella
Rothia
Streptococcus
Veillonella
Other


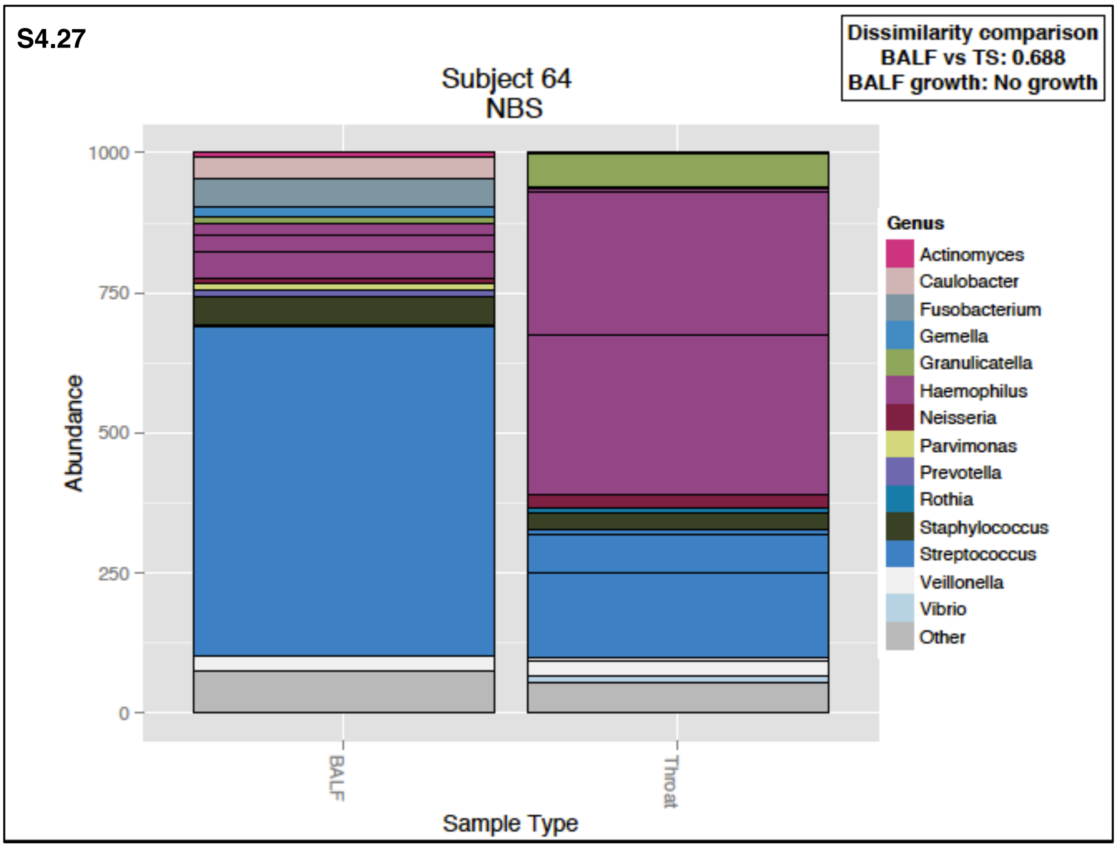

S4.27
Subject 64
NBS
Dissimilarity comparison
BALF vs TS: 0.688
BALF growth: No growth
Abundance
1000
750
500
250
0
BALF
Throat
Sample Type
Genus
Actinomyces
Caulobacter
Fusobacterium
Gemella
Granulicatella
Haemophilus
Neisseria
Parvimonas
Prevotella
Rothia
Staphylococcus
Streptococcus
Veillonella
Vibrio
Other

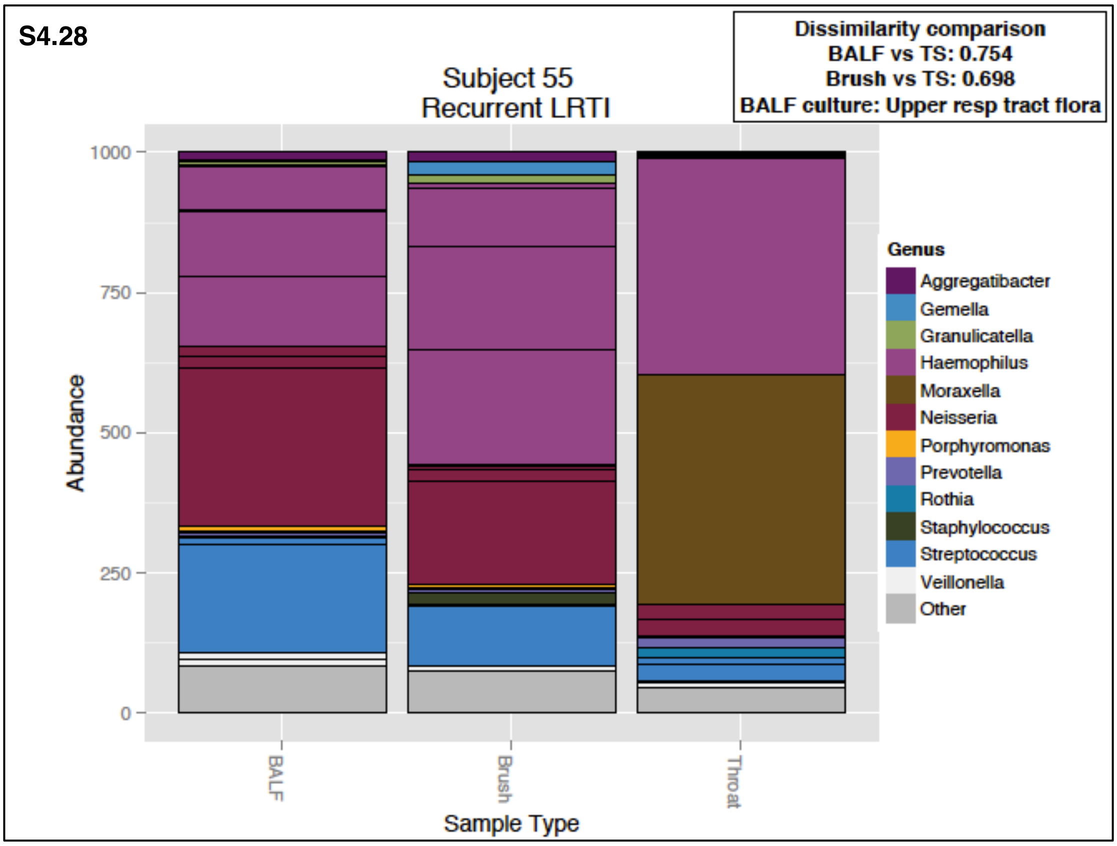
S4.28
Dissimilarity comparison
BALF vs TS: 0.754
Brush vs TS: 0.698
BALF culture: Upper resp tract flora
Subject 55
Recurrent LRTI
Abundance
1000
750
500
250
0
BALF
Brush
Throat
Sample Type
Genus
Aggregatibacter
Gemella
Granulicatella
Haemophilus
Moraxella
Neisseria
Porphyromonas
Prevotella
Rothia
Staphylococcus
Streptococcus
Veillonella
Other

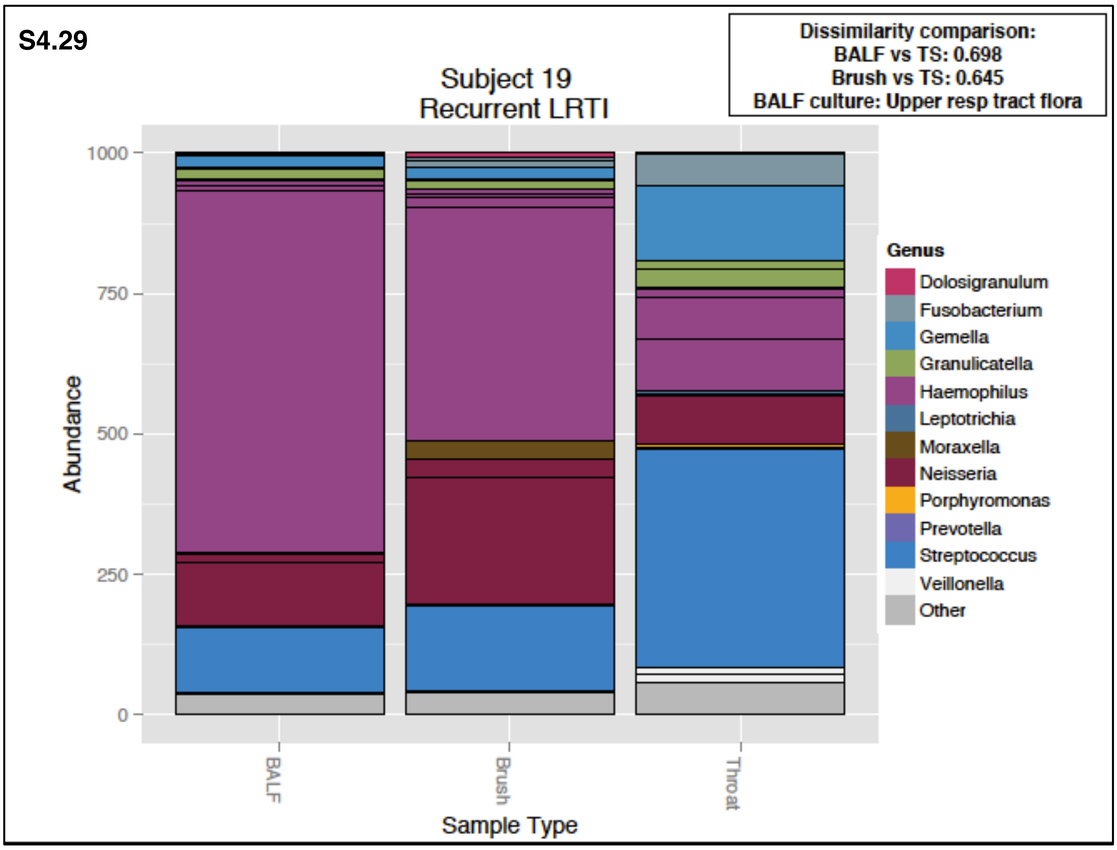
S4.29
Dissimilarity comparison:
BALF vs TS: 0.698
Brush vs TS: 0.645
BALF culture: Upper resp tract flora
Subject 19
Recurrent LRTI
Abundance
1000
750
500
250
0
BALF
Brush
Throat
Sample Type
Genus
Dolosigranulum
Fusobacterium
Gemella
Granulicatella
Haemophilus
Leptotrichia
Moraxella
Neisseria
Porphyromonas
Prevotella
Streptococcus
Veillonella
Other

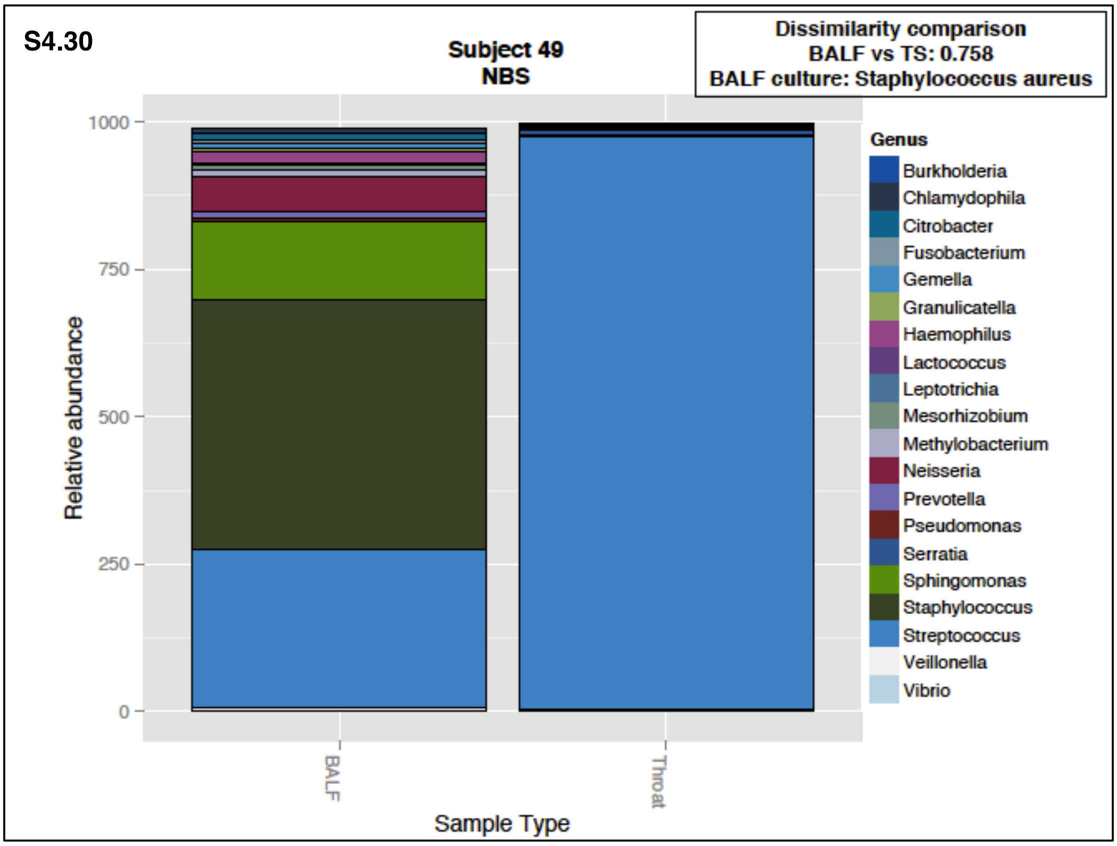
S4.30
Subject 49
NBS
Dissimilarity comparison
BALF vs TS: 0.758
BALF culture: Staphylococcus aureus
Relative abundance
1000
750
500
250
0
BALF
Throat
Sample Type
Genus
Burkholderia
Chlamydophila
Citrobacter
Fusobacterium
Gemella
Granulicatella
Haemophilus
Lactococcus
Leptotrichia
Mesorhizobium
Methylobacterium
Neisseria
Prevotella
Pseudomonas
Serratia
Sphingomonas
Staphylococcus
Streptococcus
Veillonella
Vibrio

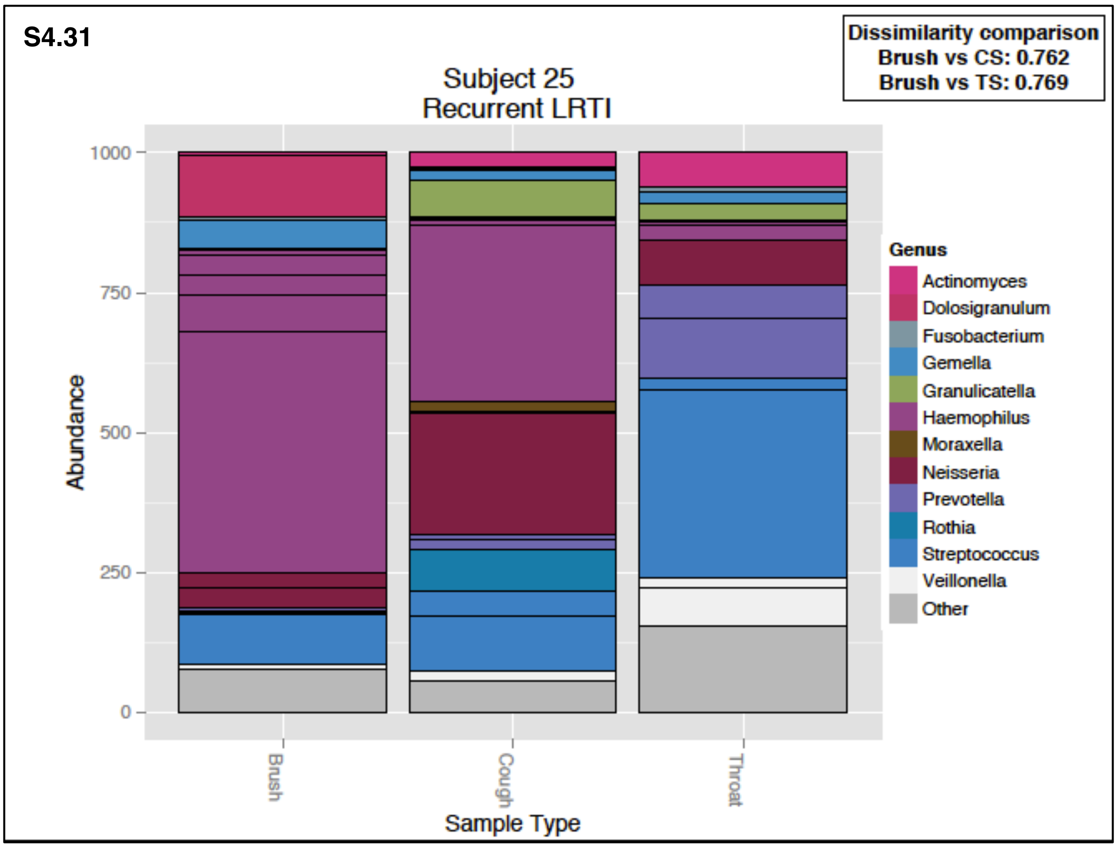
S4.31
Subject 25
Recurrent LRTI
Dissimilarity comparison
Brush vs CS: 0.762
Brush vs TS: 0.769
Abundance
1000
750
500
250
0
Brush
Cough
Throat
Sample Type
Genus
Actinomyces
Dolosigranulum
Fusobacterium
Gemella
Granulicatella
Haemophilus
Moraxella
Neisseria
Prevotella
Rothia
Streptococcus
Veillonella
Other

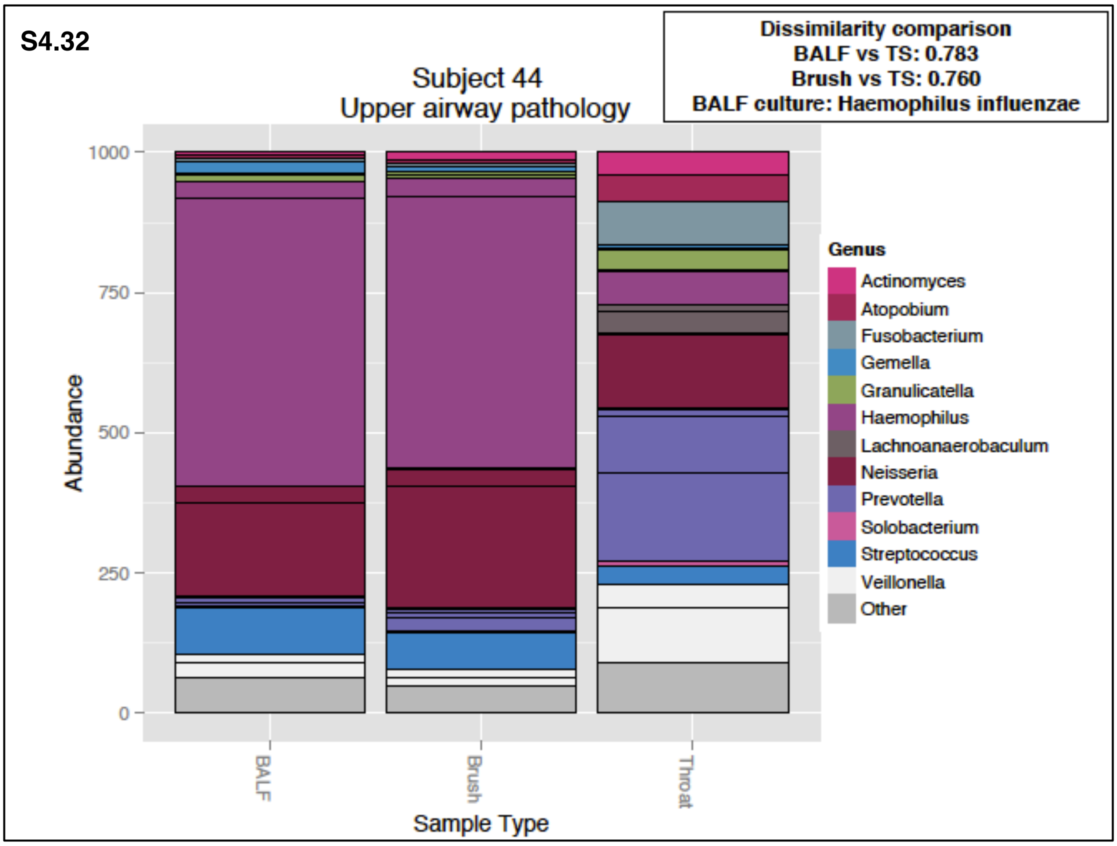
S4.32
Subject 44
Upper airway pathology
Dissimilarity comparison
BALF vs TS: 0.783
Brush vs TS: 0.760
BALF culture: Haemophilus influenzae
Abundance
1000
750
500
250
0
BALF
Brush
Throat
Sample Type
Genus
Actinomyces
Atopobium
Fusobacterium
Gemella
Granulicatella
Haemophilus
Lachnoanaerobaculum
Neisseria
Prevotella
Solobacterium
Streptococcus
Veillonella
Other

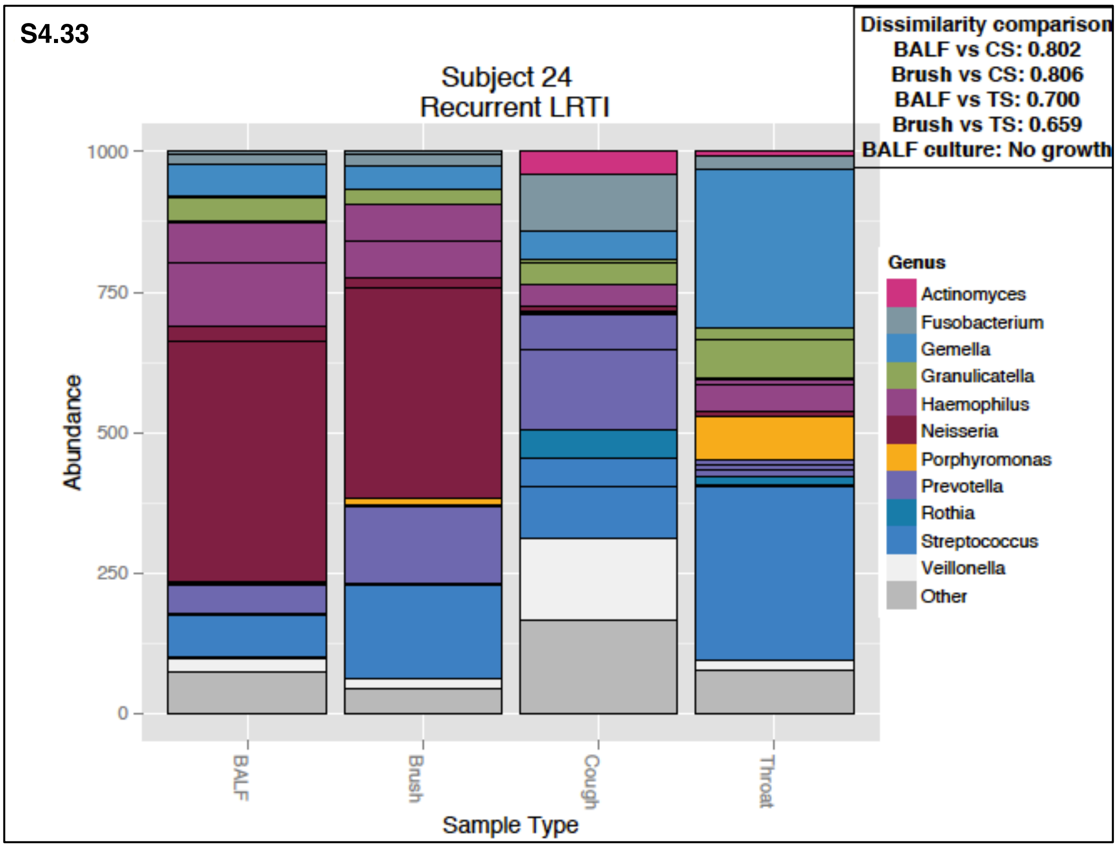
S4.33
Subject 24
Recurrent LRTI
Dissimilarity comparison
BALF vs CS: 0.802
Brush vs CS: 0.806
BALF vs TS: 0.700
Brush vs TS: 0.659
BALF culture: No growth
Abundance
1000
750
500
250
0
BALF
Brush
Cough
Throat
Sample Type
Genus
Actinomyces
Fusobacterium
Gemella
Granulicatella
Haemophilus
Neisseria
Porphyromonas
Prevotella
Rothia
Streptococcus
Veillonella
Other

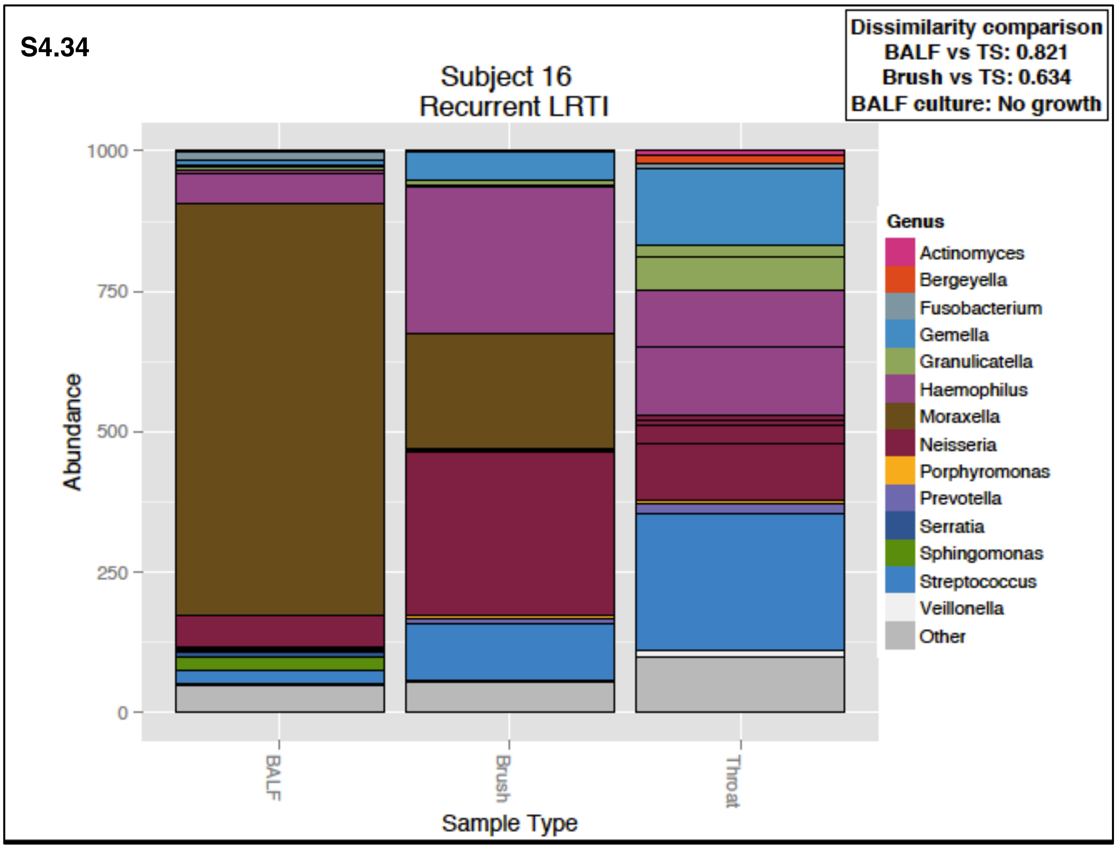
S4.34
Subject 16
Recurrent LRTI
Dissimilarity comparison
BALF vs TS: 0.821
Brush vs TS: 0.634
BALF culture: No growth
Abundance
1000
750
500
250
0
BALF
Brush
Throat
Sample Type
Genus
Actinomyces
Bergeyella
Fusobacterium
Gemella
Granulicatella
Haemophilus
Moraxella
Neisseria
Porphyromonas
Prevotella
Serratia
Sphingomonas
Streptococcus
Veillonella
Other

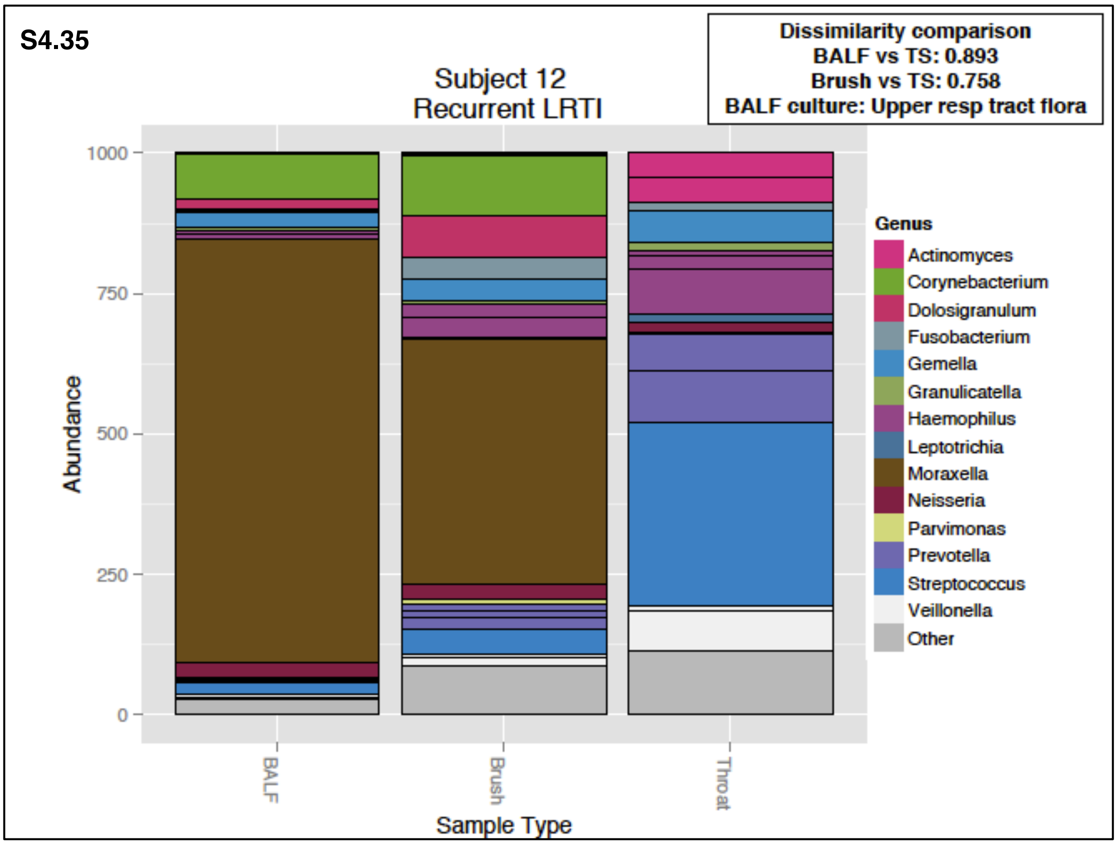
S4.35
Subject 12
Recurrent LRTI
Dissimilarity comparison
BALF vs TS: 0.893
Brush vs TS: 0.758
BALF culture: Upper resp tract flora
Abundance
1000
750
500
250
0
BALF
Brush
Throat
Sample Type
Genus
Actinomyces
Corynebacterium
Dolosigranulum
Fusobacterium
Gemella
Granulicatella
Haemophilus
Leptotrichia
Moraxella
Neisseria
Parvimonas
Prevotella
Streptococcus
Veillonella
Other

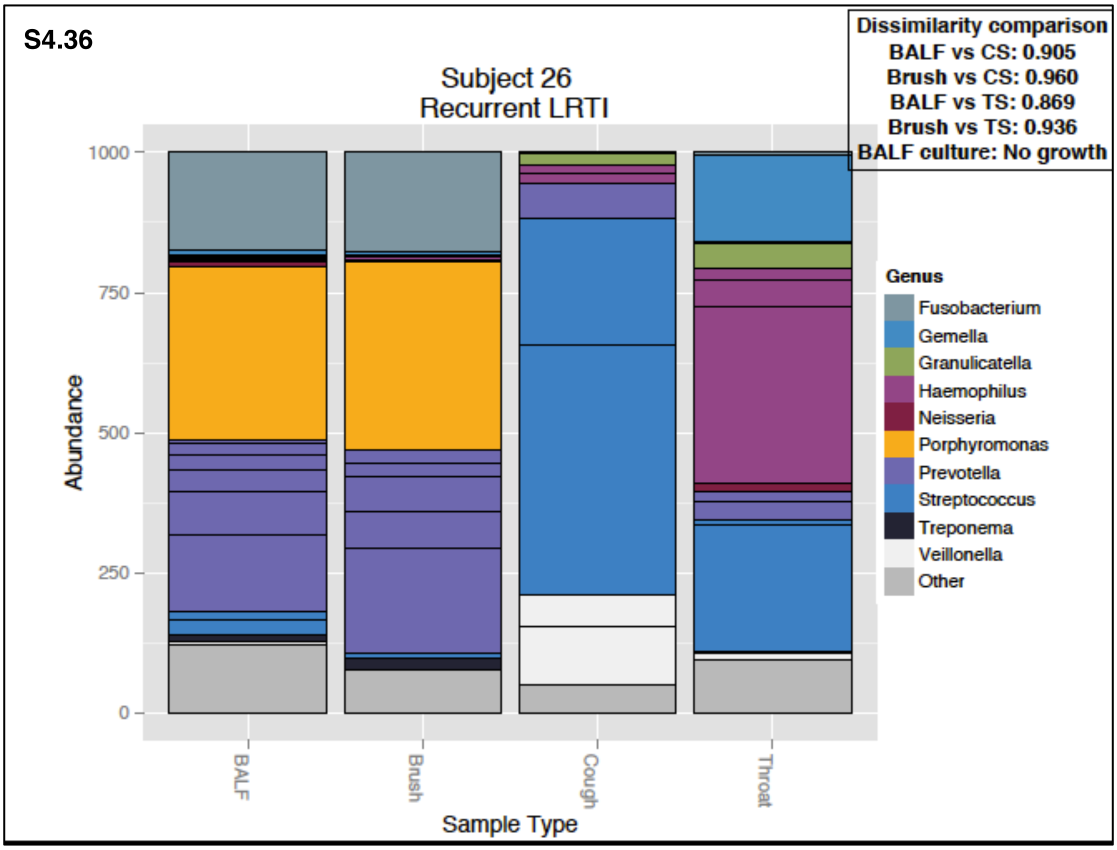
S4.36
Subject 26
Recurrent LRTI
Dissimilarity comparison
BALF vs CS: 0.905
Brush vs CS: 0.960
BALF vs TS: 0.869
Brush vs TS: 0.936
BALF culture: No growth
Abundance
1000
750
500
250
0
BALF
Brush
Cough
Throat
Sample Type
Genus
Fusobacterium
Gemella
Granulicatella
Haemophilus
Neisseria
Porphyromonas
Prevotella
Streptococcus
Treponema
Veillonella
Other

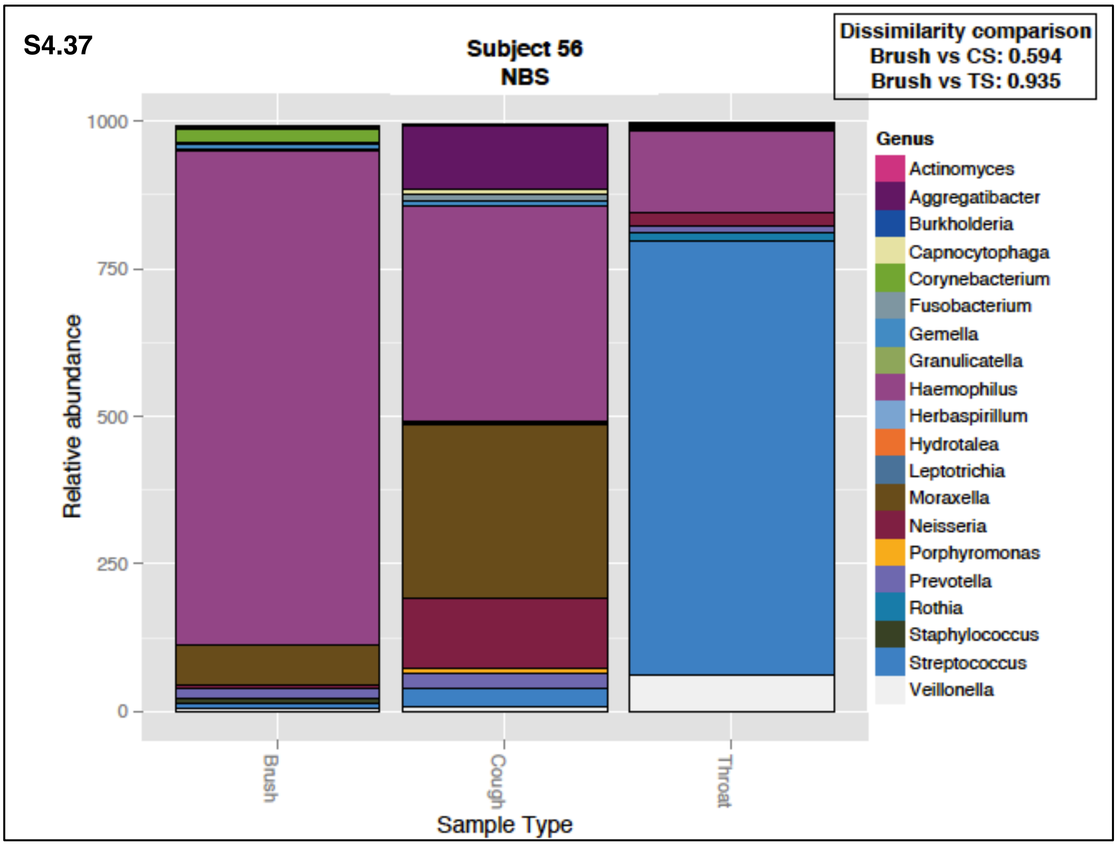
S4.37
Subject 56
NBS
Dissimilarity comparison
Brush vs CS: 0.594
Brush vs TS: 0.935
Relative abundance
1000
750
500
250
0
Brush
Cough
Throat
Sample Type
Genus
Actinomyces
Aggregatibacter
Burkholderia
Capnocytophaga
Corynebacterium
Fusobacterium
Gemella
Granulicatella
Haemophilus
Herbaspirillum
Hydrotalea
Leptotrichia
Moraxella
Neisseria
Porphyromonas
Prevotella
Rothia
Staphylococcus
Streptococcus
Veillonella

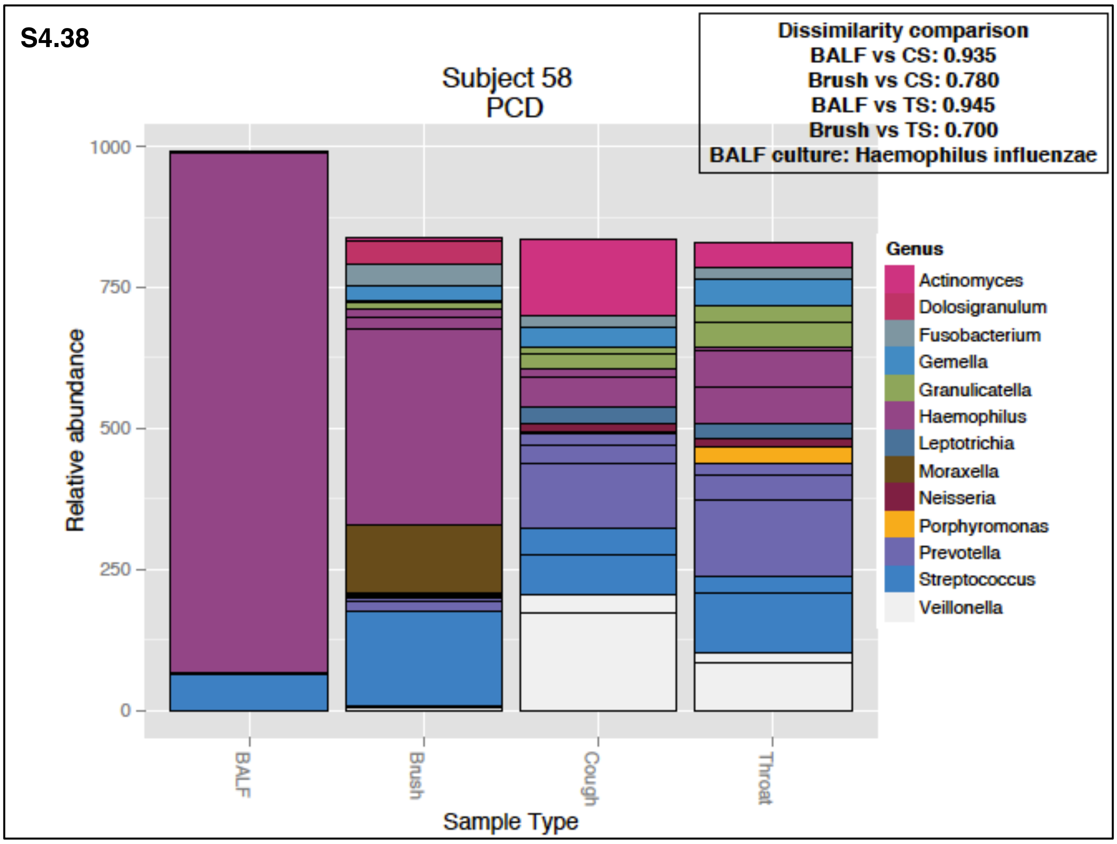
S4.38
Subject 58
PCD
Dissimilarity comparison
BALF vs CS: 0.935
Brush vs CS: 0.780
BALF vs TS: 0.945
Brush vs TS: 0.700
BALF culture: Haemophilus influenzae
Relative abundance
1000
750
500
250
0
BALF
Brush
Cough
Throat
Sample Type
Genus
Actinomyces
Dolosigranulum
Fusobacterium
Gemella
Granulicatella
Haemophilus
Leptotrichia
Moraxella
Neisseria
Porphyromonas
Prevotella
Streptococcus
Veillonella

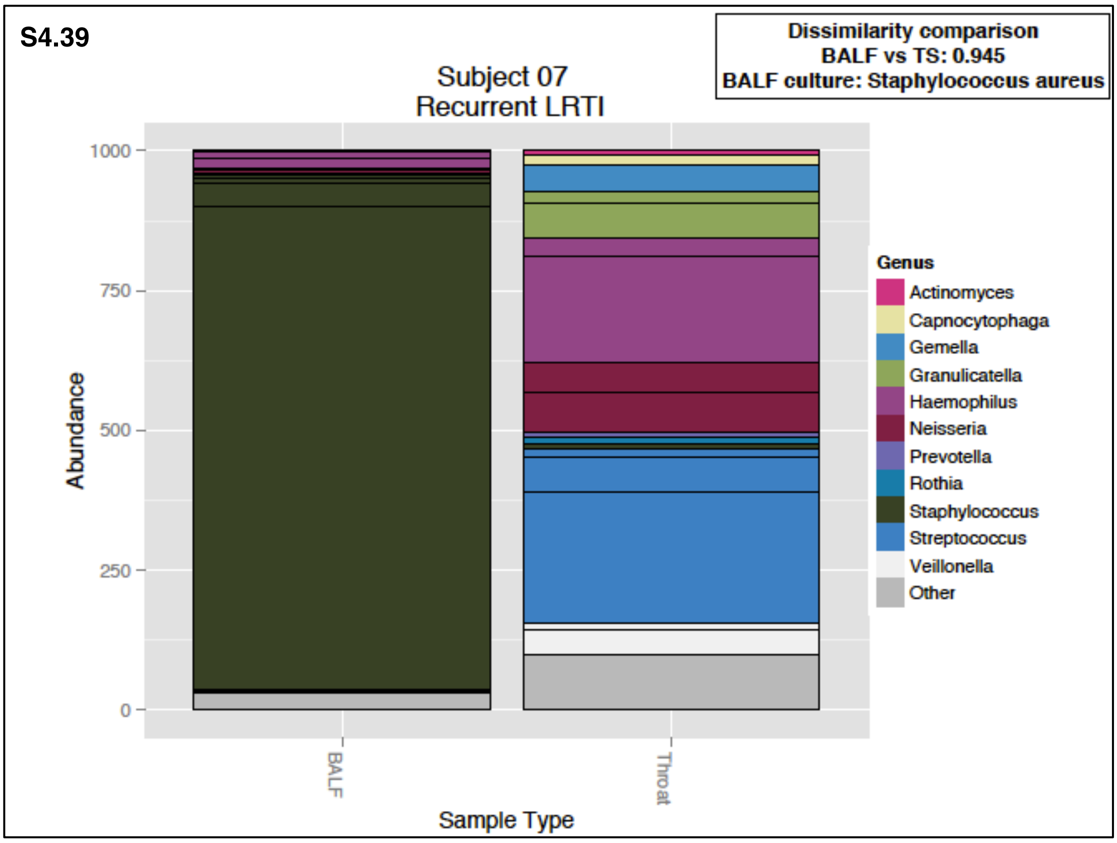
S4.39
Subject 07
Recurrent LRTI
Dissimilarity comparison
BALF vs TS: 0.945
BALF culture: Staphylococcus aureus
Abundance
1000
750
500
250
0
BALF
Throat
Sample Type
Genus
Actinomyces
Capnocytophaga
Gemella
Granulicatella
Haemophilus
Neisseria
Prevotella
Rothia
Staphylococcus
Streptococcus
Veillonella
Other

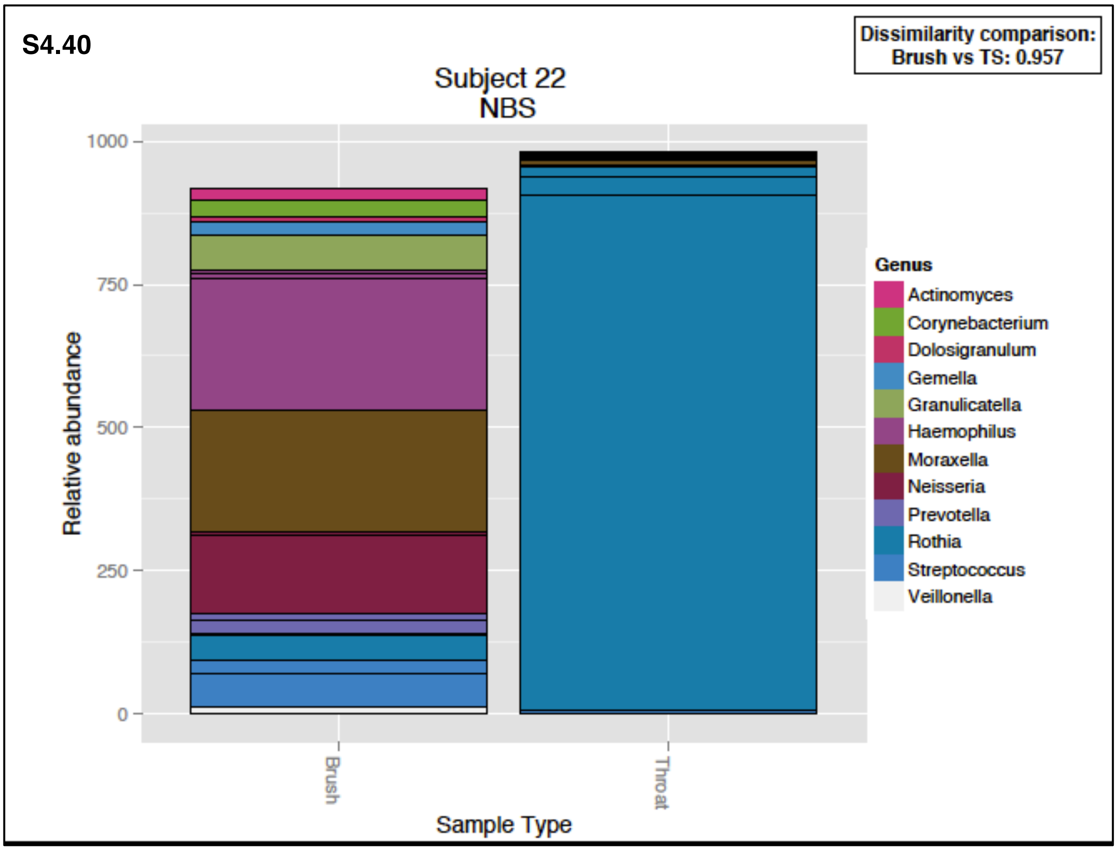
S4.40
Subject 22
NBS
Dissimilarity comparison:
Brush vs TS: 0.957
Relative abundance
1000
750
500
250
0
Brush
Throat
Sample Type
Genus
Actinomyces
Corynebacterium
Dolosigranulum
Gemella
Granulicatella
Haemophilus
Moraxella
Neisseria
Prevotella
Rothia
Streptococcus
Veillonella

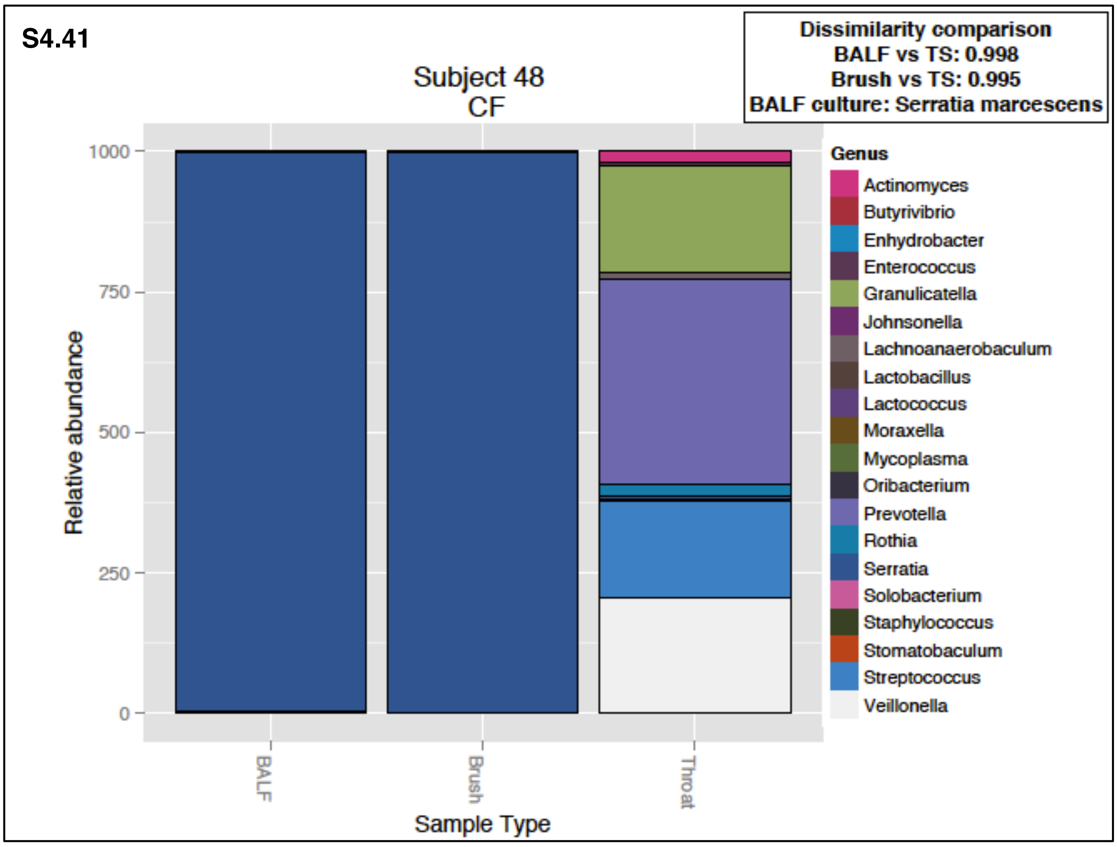
S4.41
Subject 48
CF
Dissimilarity comparison
BALF vs TS: 0.998
Brush vs TS: 0.995
BALF culture: Serratia marcescens
Relative abundance
1000
750
500
250
0
BALF
Brush
Throat
Sample Type
Genus
Actinomyces
Butyrivibrio
Enhydrobacter
Enterococcus
Granulicatella
Johnsonella
Lachnoanaerobaculum
Lactobacillus
Lactococcus
Moraxella
Mycoplasma
Oribacterium
Prevotella
Rothia
Serratia
Solobacterium
Staphylococcus
Stomatobaculum
Streptococcus
Veillonella
