# Supplementary material for: Comparison of the upper and lower airway microbiota in children with chronic lung diseases
Source: PLoS One. 2018 Aug 2;13(8):e0201156. doi: 10.1371/journal.pone.0201156 (PMC6071972; doi:10.1371/journal.pone.0201156)
Supplement: S1 Table — (DOCX) [file pone.0201156.s007.docx]

**Table S1: Summary of sequencing quality statistics**

| **Run Number** | **Number of reads**  **(in millions)** | **> Q30**  **(%)** | **Cluster density**  **(k/mm^2^)** | **Clusters passing filter (%)** |
| --- | --- | --- | --- | --- |
| BAX1 | 7.19 | 82.8 | 433 | 68.8 |
| BAX2 | 7.96 | 81.2 | 413 | 81 |
